# A Novel Role of SMG1 in Cholesterol Homeostasis That Depends Partially on p53 Alternative Splicing

**DOI:** 10.3390/cancers14133255

**Published:** 2022-07-02

**Authors:** Muyang Li, Fredrick Philantrope, Alexandra Diot, Jean-Christophe Bourdon, Patricia Thompson

**Affiliations:** 1 Department of Pathology, Renaissance School of Medicine, Stony Brook University, New York, NY 11794, USA; fphilantrope@gmail.com; 2 Division of Cancer Research, University of Dundee, Ninewells Hospital and Medical School, Dundee DD1 9SY, Scotland, UK; a.z.diot@dundee.ac.uk (A.D.); j.bourdon@dundee.ac.uk (J.-C.B.)

**Keywords:** SMG1, p53β, ABCA1, cholesterol, mevalonate, RASSF1C, miR-33a-5p, statin

## Abstract

**Simple Summary:**

p53 isoforms have been reported in various tumor types. Both p53β and p53γ were recently reported to retain functionalities of full-length p53α. A role for p53 and p53 loss in cholesterol metabolism has also emerged. We show that SMG1, a phosphatidylinositol 3-kinase-related kinase, when inhibited in p53 wild-type MCF7 and HepG2 cells, significantly alters the expression of cholesterol pathway genes, with a net increase in intracellular cholesterol and an increased sensitivity to Fatostatin in MCF7. We confirm a prior report that SMG1 inhibition in MCF7 cells promotes expression of p53β and show the first evidence for increases in p53γ. Further, induced p53β expression, confirmed with antibody, explained the loss of SMG1 upregulation of the ABCA1 cholesterol exporter where p53γ had no effect on ABCA1. Additionally, upregulation of ABCA1 upon SMG1 knockdown was independent of upregulation of nonsense-mediated decay target RASSF1C, previously suggested to regulate ABCA1 via a “RASSF1C-miR33a-ABCA1” axis.

**Abstract:**

SMG1, a phosphatidylinositol 3-kinase-related kinase (PIKK), essential in nonsense-mediated RNA decay (NMD), also regulates p53, including the alternative splicing of p53 isoforms reported to retain p53 functions. We confirm that SMG1 inhibition in MCF7 tumor cells induces p53β and show p53γ increase. Inhibiting SMG1, but not UPF1 (a core factor in NMD), upregulated several cholesterol pathway genes. SMG1 knockdown significantly increased ABCA1, a cholesterol efflux pump shown to be positively regulated by full-length p53 (p53α). An investigation of RASSF1C, an NMD target, increased following SMG1 inhibition and reported to inhibit miR-33a-5p, a canonical ABCA1-inhibiting miRNA, did not explain the ABCA1 results. ABCA1 upregulation following SMG1 knockdown was inhibited by p53β siRNA with greatest inhibition when p53α and p53β were jointly suppressed, while p53γ siRNA had no effect. In contrast, increased expression of MVD, a cholesterol synthesis gene upregulated in p53 deficient backgrounds, was sensitive to combined targeting of p53α and p53γ. Phenotypically, we observed increased intracellular cholesterol and enhanced sensitivity of MCF7 to growth inhibitory effects of cholesterol-lowering Fatostatin following SMG1 inhibition. Our results suggest deregulation of cholesterol pathway genes following SMG1 knockdown may involve alternative p53 programming, possibly resulting from differential effects of p53 isoforms on cholesterol gene expression.

## 1. Introduction

SMG1 (suppressor with morphogenetic effect on genitalia), the most recently characterized phosphatidylinositol 3-kinase-related kinase (PIKK) family member, is highly conserved across organisms [1]. Similar to structurally homologous PIKK proteins ATM and ATR, SMG1 plays a role in genome stress response (GSR) including activity to phosphorylate p53 on serine 15 [2,3,4,5]. Phosphorylation of p53 is a key step in stabilizing p53, making it more resistant to ubiquitination-mediated degradation by MDM2 [6]. SMG1 is best characterized for its role as an essential kinase in the phosphorylation of the DNA/RNA helicase UPF1, via a SMG1-SMG8-SMG9 complex, which is the first and rate-limiting step of nonsense-mediated RNA decay (NMD) [7,8,9]. NMD is a RNA quality control and gene regulatory mechanism that mediates the recognition and rapid clearance of mRNAs harboring premature termination codon (PTC) [10]. Thus, SMG1 functions uniquely in NMD and overlaps that of ATM and ATR to phosphorylate p53. With this duality of function, SMG1 has emerged in recent years as a putative tumor suppressor [4,11,12] and cancer drug target [13], though the relative importance of SMG1 in tumor biology remains unclear.

Since the late 1980s [14,15], the presence of p53 isoforms derived from alternative splicing have been observed in a variety of human cancers [16]. Experimental works support both distinct and overlapping functions of p53 isoforms with full-length p53 (p53α) [17,18,19,20]. To date, nine p53 mRNAs and at least thirteen p53 peptide variants have been reported (reviewed in [21]). On the 5′ of p53 gene, there are two promotors, P1 and P2, and two internal ribosome entry sites (IRES), ATG40 and ATG160. P1 initiates two distinct forms of p53 mRNAs, fully spliced mRNAs and intron-2 retaining mRNAs. These two types of mRNA translate into two types of N-terminal variants: the most prevalent full-length p53, which is translated from fully spliced mRNA; Δ40p53, which can be translated from ATG40 of either fully spliced mRNA or the intron-2 retaining mRNA. Both Δ133 and Δ160 are launched by P2 (which serves as introns and exons in P1-initiated mRNAs), while Δ160 begins with the alternative start codon ATG160. On the C-terminus, p53 has three distinct C-termini (Figure 2A). Despite the full-length α, β and γ, result from two ‘cryptic’ exons, 9β and 9γ, and the stop codons following them. Both 9β and 9γ have putative PTCs hypothesized as targets of NMD [22]. This is supported by Tang et al. who showed that inhibiting two NMD pathway components, SMG7 or UPF1, increased the expression of p53β [22]. More recent, Gudikote et. al. showed that pharmacologic inhibition of NMD or inhibition of UPF1 increased the expression of p53β and p53γ in MDM2 overexpressing and p53 mutant cell lines [23]. These investigators postulate that p53β and p53γ isoforms ‘restore’ p53 functionality in p53 deficient cells. However, Chen et. al. [24] provided evidence suggesting that SMG1 directly suppresses alternative splicing of p53 intron 9 by binding p53 pre-mRNA near exon 8–10. Loss of SMG1 was found to promote binding of ribosomal protein L26 (RPL26) to p53 pre-mRNA and to the recruitment of the Serine/Arginine-rich splicing factors (SRSFs), SRSF7, and expression of p53β protein. Earlier work from Tang et. al. [25] also supports a role for splicing factors. In their studies, downregulation of SRSF3 in early passage fibroblasts was mechanistically linked to alternative splicing of p53β and replicative senescence.

We are similarly interested in the effects of SMG1 on p53, p53 isoforms and their function. This includes their role in the context of work from the Prives laboratory [26,27] that identified a link between p53 and cholesterol synthesis and transport including effects on the expression of the ATP binding cassette transporter A1 (ABCA1), a transmembrane protein transporter of cholesterol [28]. The Prives group was the first to show that loss (knockout) of wild type p53 or missense mutations in p53 resulted in ABCA1 mRNA downregulation, whereas stabilization of wildtype p53 with Nutlin-3 (inhibitor of Mdm2) increased ABCA1 at the RNA and protein level. Intriguingly, loss of p53 or ABCA1 promoted sterol regulatory element-binding protein 2 (SREBP2) maturation from its uncleaved precursor and upregulation of mevalonate pathway genes involved in cholesterol synthesis. In contrast, the accumulation of wild-type p53 following Nutlin-3 treatment suppressed mevalonate (MVA) pathway signals. A follow-up study concluded that p53 inhibits cholesterol synthesis in a SREBP2 independent manner by transcriptionally suppressing squalene epoxidase (SQLE), a rate-limiting enzyme in sterol synthesis [29]. Findings from these studies have been replicated in vivo in mouse models.

On interest in SMG1, as well as NMD, as a therapeutic target for cancer [30,31] and findings that overexpression of p53β in advanced stage breast cancers is associated with better patient outcomes [32], we confirm that SMG1 knockdown increases p53β isoform protein with specific antibody. Further, we show the first evidence that SMG1 inhibition also increases transcription of the p53γ isoform. Similarly to Chen et al., we did not find an increase in p53β after targeting UPF1 for NMD-only effects. Interestingly, inhibition of SMG1, but not UPF1 in MCF7 and HepG2 cells, led to a significant alteration in cholesterol homeostasis, including increased expression of both the MVA pathway genes and ABCA1. On hypothesized effects of p53 to modulate cholesterol synthesis in tumor cells, we investigated SMG1 induced p53β and p53γ isoforms for effects on ABCA1 and cholesterol pathway genes and on the sensitivity of MCF7 to cholesterol lowering drugs following SMG1 knockdown.

## 2. Materials and Methods

### 2.1. Cell Culture and Transfection

MCF7(HTB-22), HepG2 (HB-806), and NCI-H1299 (CRL-5803) were purchased from ATCC. MCF7 was cultured in DMEM (high glucose, Gibco) with 10%FBS and 1% pen-strep. HepG2 (HB-806) and H1299 was cultured in RPMI-1640 (Gibco) with 10%FBS and 1% pen-strep. For experiments shorter than 72 h and/or with cytotoxic treatments (plasmid transfection, drug treatment, etc), cells were plated in 6-well or 24-well plates at a starting confluency of 70%. For experiments longer than 96 h (siRNA transfection), cells were plated at a lower starting confluency of 30% to prevent overconfluent.

Pre-designed siRNAs were purchased from GE Dharmacon™ (non-targeting, SMG1, GAPDH, UPF1) and Thermo Fisher Silencer Select™ (ABCA1, SREBP2, SMG1, RASSF1). Customized siRNAs of p53 isoforms were synthesized following previous publication [33]. See Supplemental List S2 for details. All siRNAs were transfected with Lipofectamine™ RNAiMAX (Thermo Fisher, Waltham, MA, USA) following official protocol.

Plasmids and plasmids/siRNA combination are transfected via Lipofectamine™ 3000 (Thermo Fisher). Due to the cytotoxicity, we used reduced amount of transfection reagent and higher seeding density. For example, to transfect MCF7 plated in 6-well plate, 6 μL reagent and 2.5 μg of DNA are used per well, and 80% confluency is required at time of transfection.

### 2.2. Cell Viability Analysis

Viability was assayed by Cell Counting Kit-8 as described by the manufacturer (CCK-8, Dojindo, Rockville, MD, USA). Briefly, CCK-8 reagent was added to cell culture medium directly and incubated for 2 h at 37 °C. Absorbance at 450 nM was measured by microplate reader. Living cell counts was determined using the linear proportional from control standard curve.

### 2.3. Western Blots

For proteins with molecular weight (MW) > 200 kDa, NuPAGE™ 3–8% tris-acetate 3–8% gels (Invitrogen, Waltham, MA, USA) and HiMark™ Pre-stained Protein Standard (Invitrogen) were used. Gels were transferred to nitrocellulose membranes via ultra-low voltage overnight transfer (12 V, ~100 mA, 20 h). For proteins with MW < 200 kDa, 10% tris-glycine gels (Invitrogen) and DualColor protein standard (Bio-rad, Hercules, CA, USA) were used instead. Primary antibodies (see Supplemental List S2 for a list of antibodies used) were diluted in 5% BSA 0.1%TBST and incubated overnight at 4 °C with gentle rocking. After primary antibody incubation, membranes were washed in 0.05% TBST three times, 10min each, followed by 1h incubation in Secondary antibody diluent. After a second series of wash, membranes were incubated in Clarity ECL substrates (Bio-rad) for 5min. Images were acquired by ChemDoc MP image system (Bio-rad).

### 2.4. Real-Time PCR and miRNA Assay

All pre-designed Taqman probes were purchased from Applied Biosciences (see Supplemental List S2 for Assay IDs). cDNA was synthesized with High-Capacity cDNA Reverse Transcription kit and RT-PCR were performed on the StepOnePlus system (Applied Biosciences, Waltham, MA, USA). All RT-PCRs were run in triplicate.

Mature microRNAs, miR-33a-5p and 3p, were first isolated by mirVana™ miRNA Isolation Kit (Invitrogen), then reverse transcribed by Taqman advanced miRNA cDNA synthesis kit (Applied Biosciences) following official protocol. Briefly, a poly-A tail and a universal 5′ adapter is ligated to 20 nt mature miRNAs sequentially, followed by reversed transcription and a miRNA-specific amplification. The RT-PCR step can be performed as standard Taqman RT-PCR using miRNA specific probes.

### 2.5. Transcriptome and Analysis

Total RNA was extracted, and RNA Integrity Number (RIN) was measured by Bioanalyzer. Library preparation (poly-A enriched, non-directional) and sequencing (20 M raw reads, paired-end 150 bp, total 6 G raw data per sample) was done by Novogene America (Sacramento, CA, USA). HISAT2 [34] was used for alignment, followed by Differential Expression Analysis (DEseq2) [35] and Gene Set Enrichment Analysis (GSEA) [36].

### 2.6. Cholesterol Staining and Analysis

To initially visualize uptake of cholesterol, a fluorescent tagged cholesterol—BODIPY-cholesterol was used. Cells were incubated in medium supplemented with 5% lipoprotein depleted serum (KalenBio, Montgomery Village, MD, USA) and 2 μM BODIPY-cholesterol (Cayman) for 48 h. Living cell fluorescent images were captured using EVOS FL Auto system (Thermo Fisher). Next, individual cells were manually outlined, and fluorescence intensity measured using the EVOS build-in software. Calculate corrected total cell fluorescence (CTCF) = Integrated Density − (Area of Selected Cell × Mean Fluorescence of Background readings).

For a more quantitative assessment, intracellular cholesterol levels were also assessed using Amplex Red by total cholesterol/total protein ratio as previously described [37]. Cells plated in 6-well plates were washed three times with PBS and then harvested in 200 μL Amplex reaction buffer (contains mild detergent) by scraping. The lysate was sonicated (5 s on and 5 s off, three cycles) to fully disrupt membranes and centrifuged at 14,000 g for 15 min. Protein concentrations were determined by Pierce BCA assay and cholesterol concentration was measured by Amplex Red cholesterol assay following official protocols.

## 3. Results

### 3.1. SMG1 Is an Unrecognized Cholesterol Metabolism Regulator

**Figure 1:**
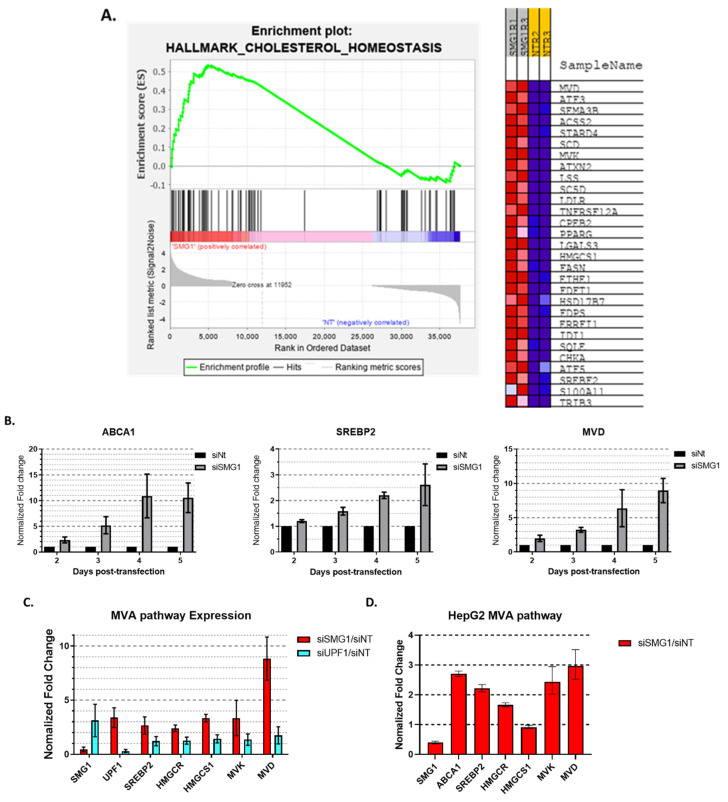
SMG1, but not NMD pathway, represses cholesterol metabolism. (**A**) Summary results of an exploratory gene set enrichment analysis from RNA-Seq data from RNA collected from MCF7 cells 5 days after transfection with SMG1 siRNA (1:1 mix of Silencer^®^ Select, s223560 and S223562, Thermo Fisher) or non-targeting siRNA (ON-TARGETplus SMARTPool of four siRNAs, GE DHARMACON). Cholesterol Homeostasis was the most enriched gene set following SMG1 knockdown (*p*-value = 0.022, FDR = 0.226); All the 29 Core Enrichment genes are listed. (**B**) Confirmational summary RT-PCR results of cholesterol pathway genes *ABCA1, SREBP2, MVD* from three independent time-course experiments (duplicated or triplicated each) of RNA collected from siSMG1 and siNT transfected MCF7 at 48-, 72-, 96-, and 120-h. (**C**) RT-PCR results for *SMG1, UPF1, SREBP2, HMGCR, HMGCS1, MVK* and *MVD* from RNA collected from MCF7 cells 5 days post-transfection with siSMG1, siNT or siRNA targeting UFP1 (ON-TARGETplus SMARTPool of four siRNAs, GE DHARMACON). Results are summary of three independent experiments (duplicated each). (**D**) RT-PCR results from a single triplicated experiment for *SMG1, ABCA1, SREBP2, HMGCR, HMGCS1, MVK* and *MVD* from RNA collected from HepG2 cells 4 days post-transfection with siSMG1 or siNT. Results in (**C**) and (D) are normalized to siNT for both siSMG1 and siUPF1 treated cells. Error bars represent Mean ± SD. For all RT-PCR studies, individual samples are run in triplicate.

**Figure 2:**
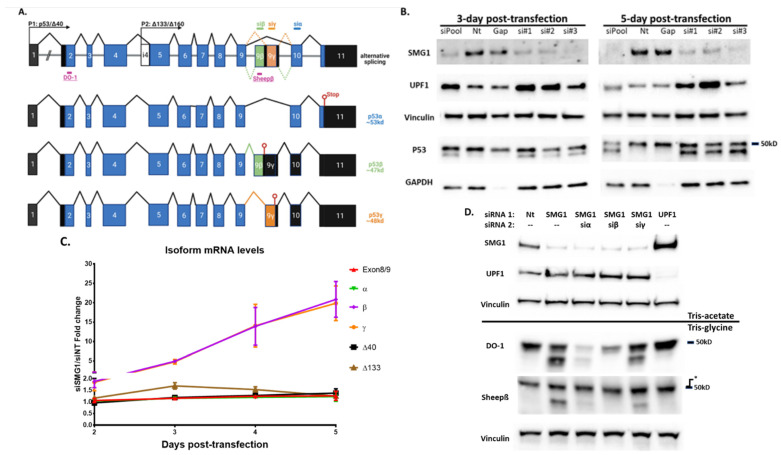
SMG1 knockdown induces p53β isoform. (**A**) Top: Schema depicting p53 alternative splicing isoforms, siRNA target exons and epitopes of p53 antibodies. Bottom: three mRNAs represent three types of C-terminus variants. Created with Biorender.com. (**B**) Proteins of p53 and p53 isoforms detected with DO-1 antibody in MCF7 p53^+/+^ cells 3 and 5 days after transfection with siRNAs. siPool and Gap, Dharmacon^TM^ ON-TARGETPlus validated 4-siRNA pool targeting SMG1 and GAPDH, respectively. NT is a 4-siRNA pool of no-target control siRNAs from the same vendor. si#1and si#2 are Silencer^TM^ select validated SMG1 siRNAs and si#3 is a 1:1 mix of si#1 and si#2. All other experiments utilized the si#3 mixture with the highest knockdown efficiency. Vinculin is the loading control. (**C**) RT-PCR analysis of p53 isoform mRNA expression in MCF7 cells following transfection with siSMG1 or siNT at 48-, 72-, 96-, and 120 h. Cells were harvested, and RNA collected at indicated times post-transfection. Taqman primers were specific to N-term variants (*Δ40* or *Δ133*) or C-term variants (*α*, *β* or *γ*) or total spliced *p53* (Exon8/9). The results of three separate experiments (duplicated or triplicated each) are summarized. Results for siSMG1 are normalized to siNT. (**D**) p53 isoform protein expression by Western blot 5 days post transfection of MCF7 cells with siRNA combinations. Results for p53 antibody DO-1 (recognizes amino acids 20-25 in exon 2) and a Sheep-host β specific antibody (recognizes amino acids peptide TLQDQTSFQKENC in exon 9β; see Appendix A Figure A2) are shown. * Asterisk indicates a known cross reactivity band that is unaffected by any p53 siRNA. Siα targets p53 mRNA exon 10, which is common in all isoforms, and mainly silences p53α; siβ and siγ targets β/γ specific c-termini, respectively (see Supplemental List S2 for sequences). * Asterisk indicates a known cross reactivity band that is unaffected by any p53 siRNA. Two types of electrophoresis gels, tris-acetate and tris-glycine were used to achieve best separation based on protein MW (see Methods 2.3 for details). Error bars represent Mean ± SD. For all RT-PCR studies, individual samples are run in triplicate. Original Western blots, view Supplemental Figures.

**Figure A1:**
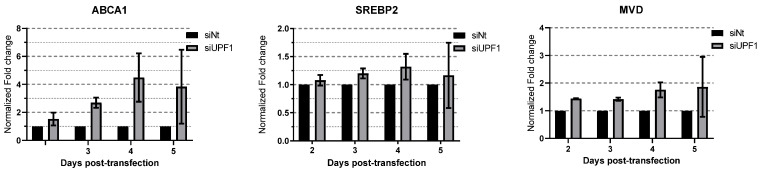
RT-PCR results of cholesterol pathway genes *ABCA1, SREBP2, MVD* from two independent time-course experiments (duplicated each) of RNA collected at 48-, 72-, 96- and 120-h post-transfection with siUPF1 and siNT. Error bars represent Mean ± SD. For all RT-PCR studies, individual samples are run in triplicate.

With interest in the global effects of SMG1 knockdown, we conducted a small-scale exploratory transcriptome investigation and discovered a previously unknown link between SMG1 and cholesterol metabolism. In gene set enrichment analysis (GSEA) (Figure 1A), SMG1 siRNA treatment of the wtp53 breast cancer cell line MCF7 results in altered expression of several genes involved in cholesterol homeostasis compared to non-target siRNA treated controls. Specifically, a number of genes involved in the MVA pathway (i.e., *MVD, MVK, LSS, HMGCS1*, and *FDFT1*), are increased in the SMG1 siRNA treated group, as seen in the DESeq2 (Supplemental List S1) and GSEA results (Figure 1A, right panel). On the contrary, cholesterol homeostasis was not among enriched gene sets in UPF1 siRNA treated cells. Follow-up confirmatory time-course gene expression profiling of two important cholesterol regulatory genes, *ABCA1* and *SREBP2*, as well as mevalonate diphosphate decarboxylase (*MVD*), a key MVA pathway enzyme gene, was also studied. *SREBP2* and *MVD* expression increased over time after siRNA transfection consistent with our GSEA exploratory results. Somewhat unexpectedly, we also observed a significant and consistent increase in the expression of the *ABCA1*, a reverse cholesterol transporter (Figure 1B). *ABCA1* expression was consistently 10-fold higher 5 days after siRNA transfection across multiple experiments. ABCA1 operates as a cholesterol pump in a cholesterol-rich environment because it transfers cholesterol from the endoplasmic reticulum as well as phospholipid from the Golgi apparatus across cell membranes. This contrasts with the increased MVA pathway (Figure 1C), which implies a low-cholesterol environment and the production of additional cholesterols. Considering that observed effects on cholesterol pathway genes may result from NMD pathway inhibition following SMG1 knockdown, UPF1 siRNA treated cells were included as NMD pathway controls. Significant inhibition of UPF1 with siRNA was confirmed by RT-PCR (Figure 1C) and Western blot (Figure 2D). UPF1 knockdown resulted in an increase in *ABCA1* (~4-fold) and *MVD* (~1.7-fold) transcripts (Appendix A Figure A1). These data support a possible role for NMD pathway inhibition in the upregulation of cholesterol homeostasis genes though the magnitude of increase was consistently less than that observed with SMG1 inhibition across multiple experiments. Notably, UPF1 knockdown reliably leads to a 3-fold increase in SMG1 expression and vice versa, at both the mRNA and protein levels (Figure 1C and Figure 2D). Knockdown of SMG1 in HepG2, a hepatocellular carcinoma cell line, resulted in similar increases in MVA pathway genes and in *ABCA1*, though to a lesser extent (Figure 1D). To our knowledge, SMG1 inhibition has not previously been link to effects on cholesterol metabolism, and the simultaneous regulation of *ABCA1* and the MVA pathway genes is inconsistent with previous work demonstrating their counter regulation in cholesterol homeostasis [26].

### 3.2. Loss of SMG1 Alternates p53 Isoform Splicing

**Figure A2:**
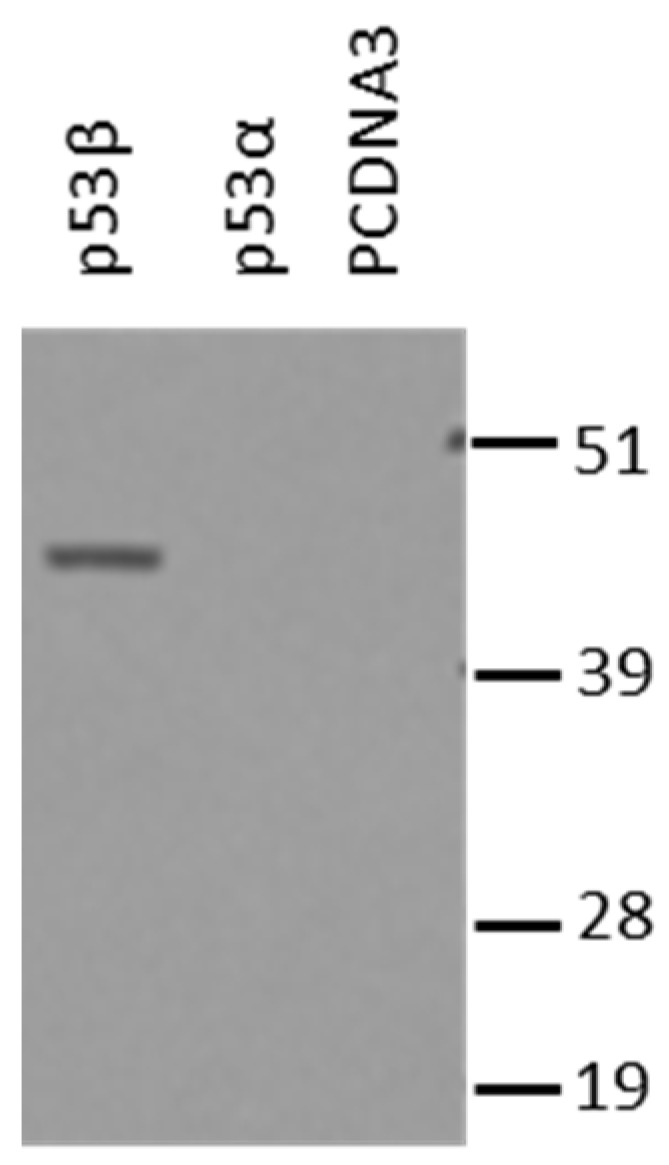
Western blot analyses demonstrating specificity of p53 specific antibody. Data are shown for H1299 p53^−/−^ cells transfected with 1 μg of pcDNA3 plasmid expressing p53β, p53α, or pCDNA3 (empty vector). Cells were harvested 24 h after transfection. The protein extract (5μg) was analyzed by Western blot using a p53β antibody raised in sheep against peptide TLQDQTSFQKENC conjugated to KLH protein. After three injections of the p53β specific peptide/KLH conjugate, serum was harvested and the p53β antibody purified by chromatography on peptide a TLQDQTSFQKENC-Sepharose bead column as previously described [19].

**Figure A3:**
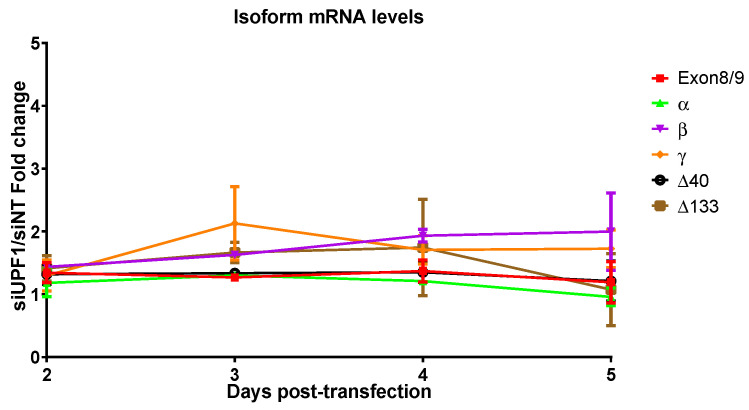
Summary RT-PCR analysis from two experiments (duplicated each) of p53 isoform mRNA expression following MCF7 transfection with siUPF1 or siNT at 48-, 72-, 96-, and 120 h. Cells were harvested at indicated time post-transfection. Results for siUPF1 are shown normalized to siNT. Taqman primers are the same as those in Figure 2B. Error bars represent Mean ± SD. For all RT-PCR studies, individual samples are run in triplicate.

Previous investigations [2,3,4,5] demonstrated that SMG1 is a classical PIKK family protein that phosphorylates p53 in response to genome stress. Additionally, a report by Chen and Kasten described a ‘SMG1-RPL26-SRSF7’ model that resulted in alternative p53 splicing and the production of the p53 C-terminal isoform, p53β [24]. Similar to their finding, using a series of siRNA combinations to knock down SMG1 and the most prevalent N-terminal antibody DO-1, we detected a putative isoform of p53 in the absence of a genome stressor (Figure 2B). To identify the specific alternative splice variants of p53 induced by SMG1 knockdown, we utilized isoform specific Taqman probes [33], isoform specific siRNAs [21] and p53 isoform antibodies (generous gifts from Dr. Jean-Christophe Bourdon, see Appendix A Figure A2 for validation) (Figure 2A). The RT-PCR results demonstrated a significant increase in the expression of *p53β* and *p53γ* mRNA following SMG1 knockdown, with > 10-fold increase in both transcripts 120 h post-transfection (Figure 2C). Furthermore, with the combination of isoform specific siRNA and isoform specific antibodies, we were able to confirm that the ‘extra’ band we observed on Western blot following SMG1 knockdown (Figure 2B) represents p53β but not p53γ (Figure 2D). We were unable to detect p53γ protein by Western blot despite significantly upregulated transcript. This may reflect the low stability of p53γ protein previously reported by Camus et al. and related to E3 ligase activity [38]. However, the use of the proteosome inhibitor MG-132 did not increase either p53β or p53γ in our studies. UPF1 knockdown (NMD control) increased *p53β* and *p53γ* mRNA level by 2- and 1.7-fold, respectively (Appendix A Figure A3) with no detectable p53β or p53γ protein (Figure 2D). Taken together, these observations support Chen and Kasten’s findings but contradict, at least partially, reports that UPF1 knockdown induces p53β at both the mRNA and protein levels [22,23], as discussed further in the discussion section.

### 3.3. P53 Isoforms Modulate the Expression of ABCA1 Differently with No Effects on SREBP2 or MVD

**Figure 3:**
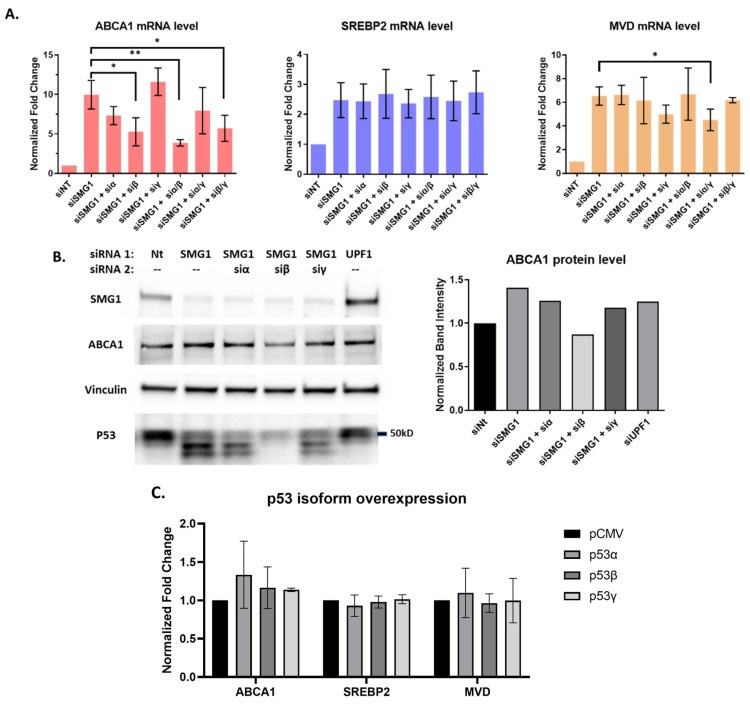
p53 isoforms differentially influence *ABCA1* expression following SMG1 knockdown but are unrelated to SMG1 effects on MVA pathway. (**A**) Summary RT-PCR results from three experiments for *ABCA1, SREBP2* and *MVD* mRNA expression from RNA collected from MCF7 cells 5 days after transfection with different siRNAs targeting SMG1 and SMG1 in combination with siRNAs targeting p53α, p53β, p53γ or the combination of p53α + p53β, p53α + p53γ, and p53β + p53γ. non-target siRNA (siNT) is included as a control. (**B**) Representative Western blot results for ABCA1 protein showing MCF7 cells treated with the different combinations of siRNAs as in panel A harvested 5 days post-transfection. The DO-1 (aa 20–25) p53 specific antibody was used examine alternative splicing. The bar plot on the right shows band intensity of ABCA1 normalized by the vinculin band intensity as a loading control. (**C**) Summary RT-PCR results from three independent experiments for *ABCA1, SREBP2*, and *MVD* mRNA expression from RNA collected from MCF7 that were harvested 2 days post-transfection with p53 isoform plasmids and compared to pCMV vector control. *p*-values shown for siSMG1 versus the different combinations of siSMG1 and p53 isoform siRNA(s) are from unpaired t-test. * = *p* < 0.05; ** *p* < 0.01. Error bars represent Mean ± SD. For all RT-PCR studies, individual samples are run in triplicate. Original Western blots, view Supplemental Figures.

Lacking a clear mechanism for SMG1 inhibition to increase both *ABCA1* expression and MVA synthesis genes, and with published studies supporting p53’s non-canonical effects on lipid and cholesterol metabolisms [39,40], including the findings from the Prives’ laboratory for a ‘p53-ABCA1-SREBP2’ metabolic axis [26], we asked if SMG1 knockdown effects on p53 isoform expression could explain any of our results. After demonstrating that SMG1 inhibition leads to an increase in p53 alternative splicing, we co-transfected MCF7 cells with SMG1 siRNA and the individual isoform-specific siRNAs and quantified *ABCA1, SREBP2*, and *MVD* mRNA levels. Under these conditions, interestingly, p53β siRNA alone or in any combination with p53α or p53γ significantly abrogated the effect of SMG1 inhibition on the upregulation of *ABCA1*, whereas p53α siRNA alone slightly reduced *ABCA1* expression (*p* = 0.1), and p53γ siRNA alone had no activity to blunt *ABCA1* transcript upregulation following SMG1 inhibition (Figure 3A). In contrast, none of the isoform specific siRNAs altered *SREBP2* or *MVD* expression, indicating that the effect of SMG1 knockdown on MVA pathway may be distinct from SMG1 effects on the expression of alternative p53 isoforms. Representative Western blot studies also show that siRNA targeting of the p53β transcript blunted the increase in ABCA1 protein expression upon SMG1 inhibition (Figure 3B), supporting the findings from the mRNA studies.

Based on these results, we investigated whether the overexpression of individual p53 isoform showed distinct regulation of *ABCA1, SREBP2*, or *MVD* expression. Using the wtp53 MCF7 cell line, we were unable to replicate earlier studies that reported direct activation of *ABCA1* following upregulated wtp53 in HCT116 and SK-HEP-1 cell lines [26]. While one or two experiments support a modest increase in ABCA1 levels following forced overexpression of p53α and p53β (no change with p53γ), the increase was not significant, and *SREBP2* as well as *MVD* mRNA levels remained unchanged (Figure 3C). To summarize, alterations in alternative splicing of p53 following siRNA knockdown of SMG1 could partially explain increased expression of *ABCA1*. The upregulation of *SREBP2* and the MVA pathway following SMG1 inhibition could not be linked to alternative splicing and p53 isoforms in MCF7.

### 3.4. miR-33a, the Canonical ABCA1 Inhibitor Is Not Perturbated by Loss of SMG1

**Figure 4:**
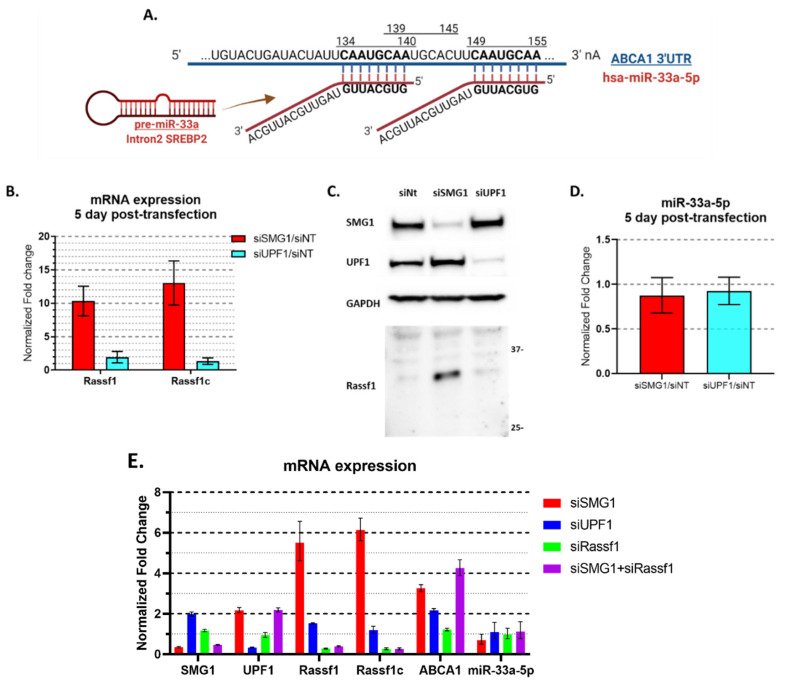
No effect of SMG1 inhibition on the expression of mir−33a−5p. (**A**) Schematic of miR−33a−5p and ABCA1 complementary sequences. miRNA−33a−5p, located in intron−2 of the SREBP2 gene, is complementary to 3’ UTR of ABCA1 and inhibits ABCA1 expression [41,42,43,44]. Created with Biorender.com. (**B**) Summary RT-PCR results from three experiments (duplicated each) for RASSF1 and RASSF1C mRNA levels in MCF7 cells 5 days post transfection with siSMG1, siUPF1 or siNT. (**C**) Western blot results for RASSF1C (probed with Rassf1 [EPR7127], Abcam; see Appendix A Figure A4 for validation) on Western blots of a representative experiment showing MCF7 cells treated with different siRNAs. Cells are harvested 5 days post-transfection. (**D**) Summary RT-PCR for two experiments (duplicated each) for miR−33a−5p from RNA collected from MCF7 cells 5 days post transfection with siRNA targeting SMG1, UPF1 and non-target controls. miR−33a−5p cDNA were synthesized via TaqMan™ Advanced miRNA cDNA Synthesis Kit (Applied Biosystems), probed by TaqMan Advanced miRNA Assays and normalized by miR−16−5p. (**E**) Representative RT-PCR mir−33a−5p results for a single triplicated experiment for MCF7 cells 3 days post-transfection with siSMG1, siUPF1, siRASSF1 or the combination of siSMG1 and siRASSF1. Error bars represent Mean ± SD. For all RT-PCR studies, individual samples are run in triplicate. Original Western blots, view Supplemental Figures.

**Figure A4:**
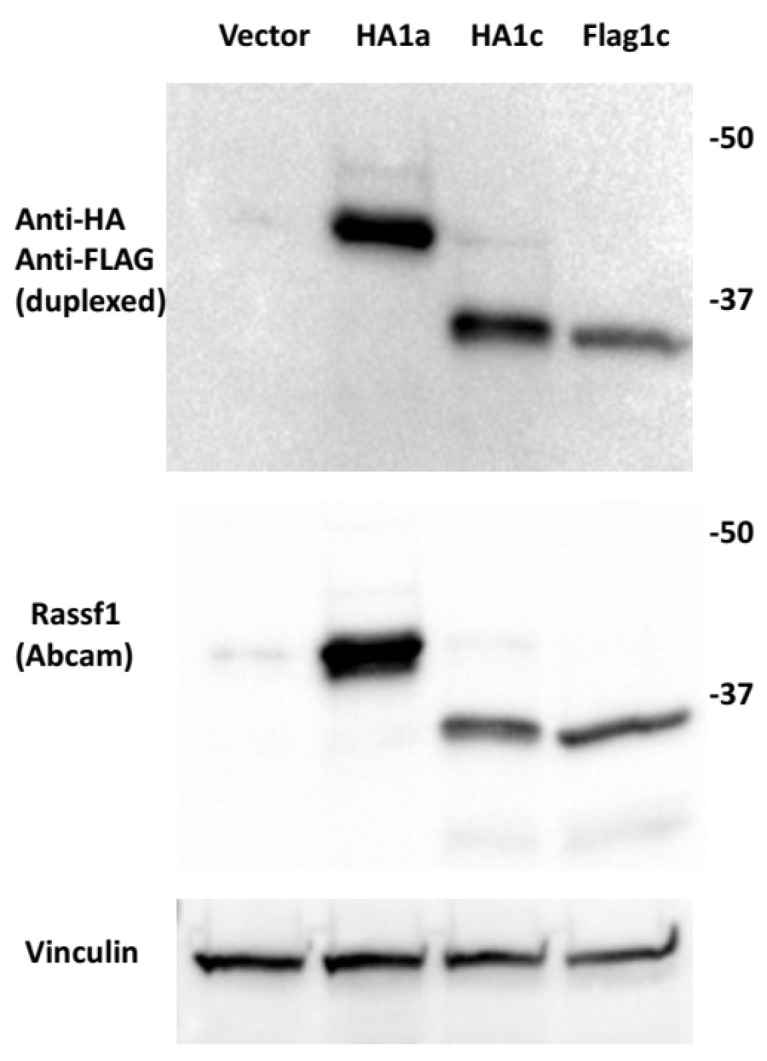
Western blotting verification of RASSF1 antibody and overexpressed isoforms. H1299 cells were transfected with HA-tagged RASSF1A (Addgene#1980), HA-tagged RASSF1C (Addgene#1981), FLAG-tagged RASSF1C (Addgene#1977) or empty vector and harvested 2 days post-transfection. HA or FLAG tags were probed by Anti-HA: Anti Flag mixed antibodies. RASSF1A and C were probed by RASSF1 antibody (EPR7127, Abcam). Vinculin was probed as loading control.

In canonical models of *ABCA1* gene regulation [41,42,43,44], microRNA-33a-5p, embedded in the *SREBP2* gene intron-2 and complementary to *ABCA1* 3′ untranslated region (3′ UTR), inhibits *ABCA1* expression (Figure 4A). While no link between SMG1 and miR-33a-5p has previously been reported, a recent study implicated the putative NMD target, Ras association domain-containing protein 1C (*RASSF1C*) as an inhibitor of miR-33a-5p [45]. Thus, we hypothesized that NMD inhibition following SMG1 silencing may result in an increase in RASSF1C and act on ABCA1 via miR-33a-5p inhibition. RASSF1C mRNA and protein levels were found to increase significantly as expected in response to SMG1 knockdown in MCF7 cell (Figure 4B,C). *RASSF1C* mRNA increased > 10-fold 5 days following transfection with siRNA targeting SMG1, while only slightly increased (1.4-fold) with UPF1 siRNA (Figure 4B). In line with the mRNA expression, SMG1 knockdown, but not UPF1 knockdown, induced a band on Western blot at the expected molecular weight detected by RASSF1 antibody (Figure 4C; see Appendix A Figure A4 for antibody validation). These data show that *RASSF1C* upregulation in MCF7 cells was specific to SMG1 knockdown and not observed with UPF1 inhibition despite clear inhibition of UPF1 at the protein level.

**Figure A5:**
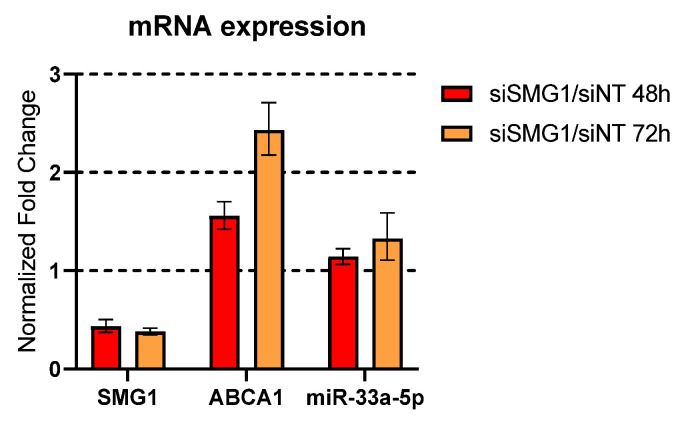
Representative RT-PCR results for *SMG1, ABCA1* and miR-33a-5p expression levels in RNA collected from MCF7 cells from a single triplicated experiment 48- and 72-h post-transfection. Error bars represent Mean ± SD. For all RT-PCR studies, individual samples are run in triplicate.

Mature microRNAs are short (22 nt) single-stranded non-coding RNAs that act as an active component of the RNA interfering mechanism. These mature microRNAs are difficult to stabilize and cannot be probed using conventional RT-PCR. We utilized a workflow specifically designed for mature microRNAs (see Methods) to probe the *ABCA1* inhibitor miR-33a-5p. Initially, we measured miR-33a-5p levels 5 days after siSMG1 or siUPF1 transfection and found no significant changes (Figure 4D). Given the possibility that miRNA disruption may occur earlier post-transfection, we next validated that the levels of miR-33a-5p were unaffected 2 or 3 days after transfection of MCF7 cells with SMG1 siRNA versus control siRNA (Appendix A Figure A5), indicating that the increase in *RASSF1C* was not associated with a decrease in miR-33a-5p as previously reported [45]. To explore this further in our model on concerns over the detectability of miR-33a-5p, we more directly examined the activity of RASSF1C on *ABCA1* expression in MCF7. As reported by others [46,47], *RASSF1A*, the major isoform of *RASSF1C*, is silent in most cancer cell lines and our RT-PCR trials cannot detect its expression in MCF7, H1299, or HepG2 (failed to amplify under any circumstances). Thus, we used a RASSF1 siRNA that targets a single exon shared by all RASSF1 isoforms. The RASSF1 siRNA successfully reduced total *RASSF1* and *RASSF1C* levels but showed no effect on *ABCA1* expression or on miR-33a-5p levels either alone or in combination with SMG1 inhibition with siRNA (Figure 4E). These data show that NMD inhibition via SMG1 knockdown results in increased RASSF1C in MCF7 but that the increase in *ABCA1* transcription could not be explained by any effect of RASSF1C via inhibitory activity on miR-33a-5p.

### 3.5. Loss of SMG1 Increased Intracellular Cholesterol Level

**Figure 5:**
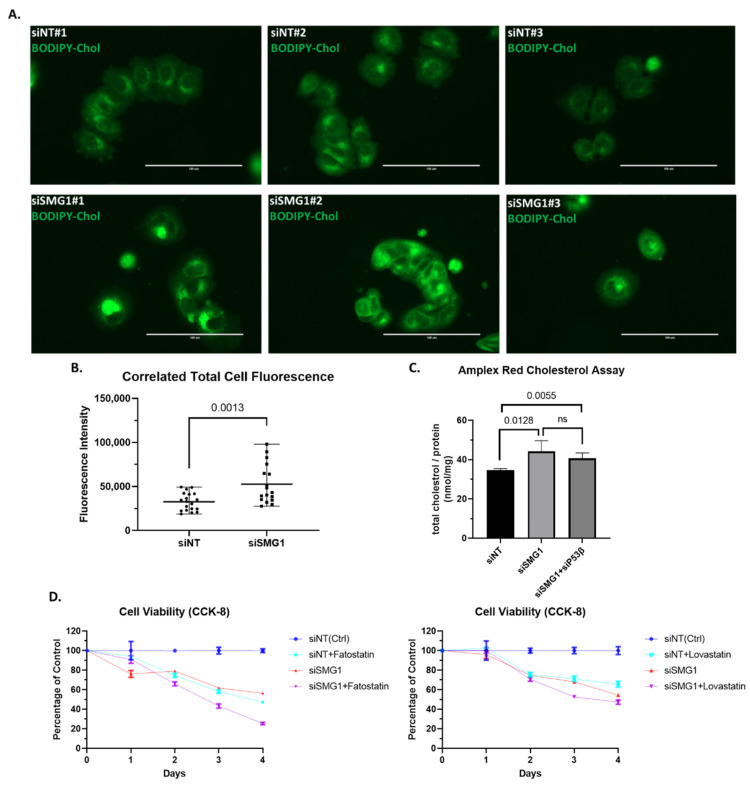
SMG1 knockdown increases intracellular cholesterol levels and sensitizes MCF7 cells to Fatostatin, and less degree to Lovastatin. (**A**) Representative images of the immunofluorescence staining of BODIPY-cholesterol in MCF7 grown in 35mm glass bottom dishes. Images were acquired 4 days post-transfection with siSMG1 or siNT, using an EVOS FL Auto Fluorescence Inverted Microscope Imaging System. For each siRNA treatment group, three randomly selected areas of interest are shown (Bottom right scale bar, 100μM). (**B**) Captured images are summarized as a corrected total cell fluorescence (CTCF) statistic. Intensities of cells, as well as background intensity were measured by EVOS software. CTCF = cell intensity − (background intensity × area of outlined cell). 19 cells in siNT group, and 17 cells in siSMG1 group were counted. Presents Error bar shows Mean with Range. (**C**) Total intracellular cholesterol level was quantified in MCF7 cells 4 days post-transfection with siRNA targeting NT, SMG1 and SMG1 + p53β by Amplex Red cholesterol assay; four replications per treatment group. Total protein, measured by Pierce BCA assay, was used as an input reference (see Method 2.6 for details). ns, not significant. (**D**) To assess SMG1 inhibition effects on MCF7 sensitivity to cholesterol lowering agents, MCF7 cells were plated in 24-well plates and transfected 24 h later with siRNA targeting NT or SMG1 and then treated with Fatostatin (25 μM), Lovastatin(25 μM) or DMSO (control) as indicated. Each treatment group was performed in triplicate. Post-treatment viability was assessed every 24 h using the colorimetric CCK-8 cell viability assay until posttreatment day 4.

Having consistently observed upregulation of *ABCA1,* a reverse cholesterol transporter, and MVA synthesis genes following SMG1 knockdown, we examined the more direct effect of SMG1 inhibition on intracellular cholesterol levels. Following siRNA transfection, MCF7 cells were cultured in serum depleted of lipoproteins and supplemented with fluorescent BODIPY-cholesterol. After 2 days, the fluorescent cells were observed via fluorescence microscopy. In comparison to non-targeting siRNA treated control cells, which exhibited a typical MCF7 flat-sheet morphology, SMG1 siRNA targeted cells had a rounded shape with less extracellular matrix (Figure 5A). Additionally, SMG1-siRNA targeted cells exhibited significantly increased fluorescent signal confirmed by corrected total cell fluorescence (CTCF) analysis (Figure 5B). To confirm and quantify the apparent increase in intracellular cholesterol level observed with image analysis following SMG1 knockdown, we utilized an Amplex Red cholesterol fluorometric method to quantify both free and esterified cholesterol (or total cholesterol). The cholesterol values were normalized against the protein concentration, yielding a cholesterol-to-protein ratio, as previously described [37]. In line with the CTCF analysis, SMG1 siRNA treated cells had higher level of intracellular cholesterol than non-targeted siRNA treated cells (Figure 5C). However, a combination of SMG1 and p53β siRNAs did not significantly differ from SMG1 siRNA alone, implying that changes in *ABCA* levels may not have a direct effect on cholesterol levels. Combining results from these two methods, we concluded that inhibition of SMG1 results in an increase in intracellular cholesterol levels, despite the 10-fold increase in *ABCA1* gene expression.

Given that loss of SMG1 expression results in an increase in intracellular cholesterol levels, we wish to establish whether this increases cancer cell sensitivity to inhibitors of cholesterol synthesis. To our surprise, MCF7 cells treated with siRNA to SMG1 were more sensitive to the growth inhibitory effects of Fatostatin, an inhibitor of SREBP activation, than to Lovastatin, a clinically approved HMG-CoA reductase inhibitor (Figure 5D). Fatostatin is known as a specific inhibitor of SREBP cleavage-activating protein (SCAP), a required protein for SREBP activation, but also has SREBP-independent effects [48,49]. This raises the possibility that the observed co-efficiency of Fatostatin and SMG1 knockdown is due to factor other than an SREBP2–MVA pathway.

## 4. Discussion

Here, we confirm that inhibiting PIKK SMG1, but not the core NMD factor UPF1, in p53 wildtype MCF7 mammary cells induces the expression of p53β with the first evidence for concomitant upregulation of p53γ. Coincident with SMG1 knockdown and alternative splicing of p53, we demonstrate a significant upregulation in cholesterol synthesis gene expression not observed with UFP1 targeting. We show that the effects of SMG1 knockdown on cholesterol pathway genes is explained by the upregulation of the p53 exon 9 isoforms including an increase in *ABCA1* following the expression of p53β. Noting that Amaar and Reeves recently reported [45] that *RASS1C*, a known NMD target upregulated by SMG1 knockdown, has activity to inhibit miR-33a-5p, a validated microRNA and canonical inhibitor of *ABCA1* housed in intron 2 of the *SREBP2* gene [41,42,43,44], we examined this alternative mechanism but found no evidence to support a role for the NMD target RASSF1C in *ABCA1* expression.

Like Chen et. al., [24] our results indicate that SMG1 loss significantly alters the expression of p53 isoforms from exon 9 and alters the expression of p53 target genes. Our work extends the effect of SMG1 loss to alterations in the expression of several cholesterol pathway genes mediated in part through upregulation of p53β and p53γ isoforms. One unexpected observation was the concurrent upregulation of both *ABCA1* and *SREBP2-MVA* mRNA upon SMG1 knockdown. In earlier work, ABCA1 has been identified as positively regulated by p53 [26] while MVA pathway genes have been shown to be upregulated in p53 null or mutant backgrounds [27] with some, but not all, studies showing dependence on SREBP2 [29]. We suspect that the concurrent increase in cholesterol transport genes and MVA synthesis following SMG1 loss reflects deregulation of p53 target gene control mediated through alternative splicing and altered function of p53αThe observation that p53β is mediating the increase in *ABCA1* following inhibition of SMG1; a function ascribed to full-length p53 [26]. The activity of p53β on *ABCA1* supports Heymach [23] premise that the exon 9 derived isoforms share overlapping functionalities with full-length p53α to compensate for p53 loss. This idea is supported by Chen et al. [24] who demonstrated that p53β when overexpressed had activity to regulate subsets of p53 target genes some independent of and some dependent on the presence of p53α. Our story with p53γ, which could not be detected on Western blot presumably a result of lower stability [38], is less clear. Induction of p53γ appears to explain the effects of SMG1 inhibition to increase MVD (MVA pathway gene) but showed no activity to increase *SREBP2* expression. This result suggests that p53γ activity is distinct from p53β. Notably, our data also do not support a role for any of the p53 isoforms in the increase in expression of the *SREBP2* gene following SMG1 inhibition.

Importantly, and a limitation of our study, the mRNA for p53α does not contain a p53α ‘specific’ sequence for exclusive siRNA targeting. In previous studies [21] and in these new results, targeting of exon 10 with siRNA shows preferential activity to inhibit p53α and thus, siRNA targeting exon 10 is commonly referred to as a siRNA to p53α. In contrast, selective siRNAs can be, and have been designed and validated, against exon 9-9β for p53β silencing and exon 9-9γ for p53γ silencing [21]. Exon 10 however is also part of the 3′ untranslated region of p53β and p53γ. Theoretically, siRNA targeting of exon 10 could inhibit the expression of p53β and p53γ. Indeed, when we ectopically express p53β and p53γ both are slightly decreased with siRNA targeting exon 10. We interpret these results cautiously as evidence that the siRNA targeting of exon 10 predominantly suppresses p53α with slight inhibition of p53β and p53γ. Thus, in Figure 3A,B, the effect of siα (siRNA to exon 10) to reverse the effect of SMG1 knockdown on ABCA1 could result from inhibiting p53α alone, p53β, or both. Because we observed that the combined treatment of siRNA to exon 10 (siα) and siRNA to p53β (siβ) blunted the effect of SMG1 inhibition to a greater extent on ABCA1 expression (and siα and siγ to blunt the activity of SMG1 inhibition to increase MVD), our main conclusions are that p53β has activity to target gene ABCA1 and p53γ to impact MVD and that their activity on these gene targets likely depends on p53α. This is consistent with findings that p53β impacts the expression of a subset of p53 target genes in ‘collaboration’ and not through physical contact with p53α [24].

Observing an increase in intracellular cholesterol and altered p53 expression, we wanted to know if loss of SMG1 increased the sensitivity of MCF7 to cholesterol lowering drugs similar to reports for p53 null/mutant cells [26,27]. SMG1 knockdown appeared to sensitize MCF7 cells more to Fatostatin, an inhibitor of SREBP activation, than to Lovastatin, a competitive inhibitor of 3-hydroxy-3-methylglutaryl-coenzyme A (HMG-CoA) reductase and the rate-limiting enzyme in cholesterol synthesis. Because Fatostatin has been reported to have non-selective activity to inhibit endoplasmic reticulum to Golgi transport [49], it is possible that increased sensitivity to Fatostatin is related to the unfolded protein response induced by inhibiting NMD [50]. Additional work is needed to separate p53 deregulation from NMD in response to lipid-lowering drugs following SMG1 inhibition.

Despite the observation that p53 isoforms are differentially expressed in development and in different organs, tissue types and in human tumors, their study has received limited attention. This reflects several technical challenges. The widely used DO-1 antibody can detect only one of the four N-terminal variants, and any C-terminal variants detected are widely ignored as “background” “shades” or “doubling bands” due to molecular weights close to p53α (full-length p53). While Fahraeus (Δ40p53), Harris (Δ133p53), Lane and Bourdon (all p53 isoforms), as well as several reports by others evaluating expression in different tumor types (reviewed in [16]) have provided some initial insights about p53 alternative splicing and biological effects, the role and control of p53 isoforms in normal and in tumor biology remain poorly understood. Several commercially available p53 antibodies targeting various epitopes have been discontinued. Antibodies specific for p53 isoforms are difficult to develop due to near identical amino acid sequences. Both the C-terminal variants, β and γ, have very short unique peptide sequences, 10 and 15 amino acids, respectively. These C-termini are also actively post-translationally modified, making it more difficult to develop high-quality specific antibodies. With evidence that p53β and p53γ impact the expression of p53 target genes including cell metabolism, a significant need exists for isoform specific tools and model systems to better define the role of p53 isoforms in cellular functions remains.

## 5. Conclusions

Our studies support findings that SMG1 is acting as a major regulator of p53 exon 9 isoform expression [24,32], and the studies of Bourdon and others [19,51] that the gene targets and cellular outcomes of ‘p53′ are determined by a more complex complement of p53 isoforms than what is currently understood. Our results demonstrate a role for p53β in the upregulation of ABCA1 following SMG1 inhibition with evidence that p53γ may explain increases in MVA pathway genes following loss of SMG1. Similarly to Chen et al., [24] we find strikingly different effects of SMG1 inhibition on the p53 exon 9 isoforms in MCF7 cells, compared to reported UPF1 inhibition in other cell lines [22,23]. One possibility is that, despite high efficiency inhibition of UPF1, the NMD pathway in MCF7 is not completely inhibited by targeting UPF1 alone. This is interesting as it agrees with the findings from Heymach [23] whose has suggested that ‘p53 deficiency’ increases the sensitivity of cell lines to NMD inhibitors as a druggable vulnerability. Our work partly supports the premise that p53β and p53γ isoforms may partly restore p53 functionality in a p53 deficient state though in balance, following SMG1 inhibition MCF7 cells have increased intracellular cholesterol and are more vulnerable to lipid lowering drugs more like p53 null or mutant cells. Importantly, with the broadening interests in NMD [23] and SMG1 [13] as drug candidates, a better understanding of the regulation and function of p53 and p53 isoform will be important for predicting the consequences of targeted interventions on these pathways.

## Data Availability

The data presented in this study are available in the article and Supplementary Materials.

## Supplementary Materials

The following supporting information can be downloaded at: https://www.mdpi.com/article/10.3390/cancers14133255/s1, Supplemental List S1: DEseq2 results; Supplemental List S2: Material List. Supplemental Figures: original Western blots.
