# Supplementary material for: A Novel Role of SMG1 in Cholesterol Homeostasis That Depends Partially on p53 Alternative Splicing
Source: Cancers (Basel). 2022 Jul 2;14(13):3255. doi: 10.3390/cancers14133255 (PMC9265556; doi:10.3390/cancers14133255)
Supplement: Supplementary file 1 [file cancers-14-03255-s001.zip › Supplemental data/Supplemental Figures (original Western blots).pdf]

Original Western blots

Whole blots/strips captured in ChemiDoc Imaging Systems are shown, density of bands (relative to control band) annotated.

Figure 2B.

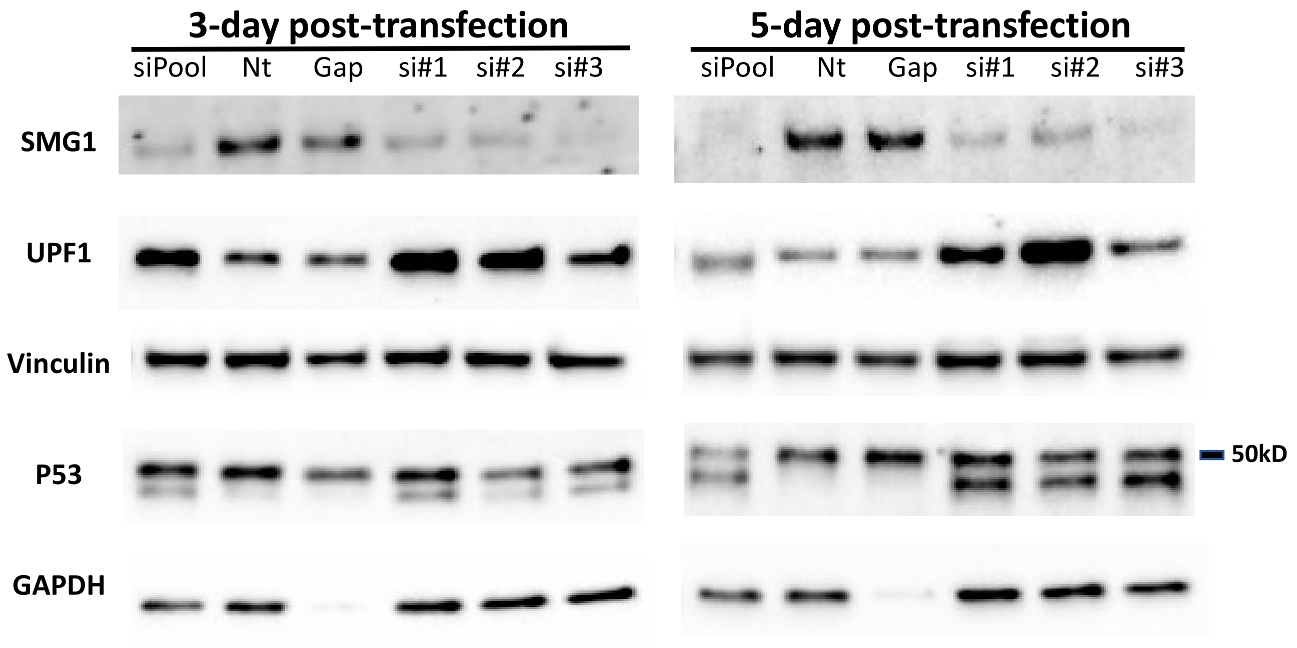

3-day post-transfection
siPool
Nt
Gap
si#1
si#2
si#3
5-day post-transfection
siPool
Nt
Gap
si#1
si#2
si#3
SMG1
UPF1
Vinculin
P53
GAPDH
50kD

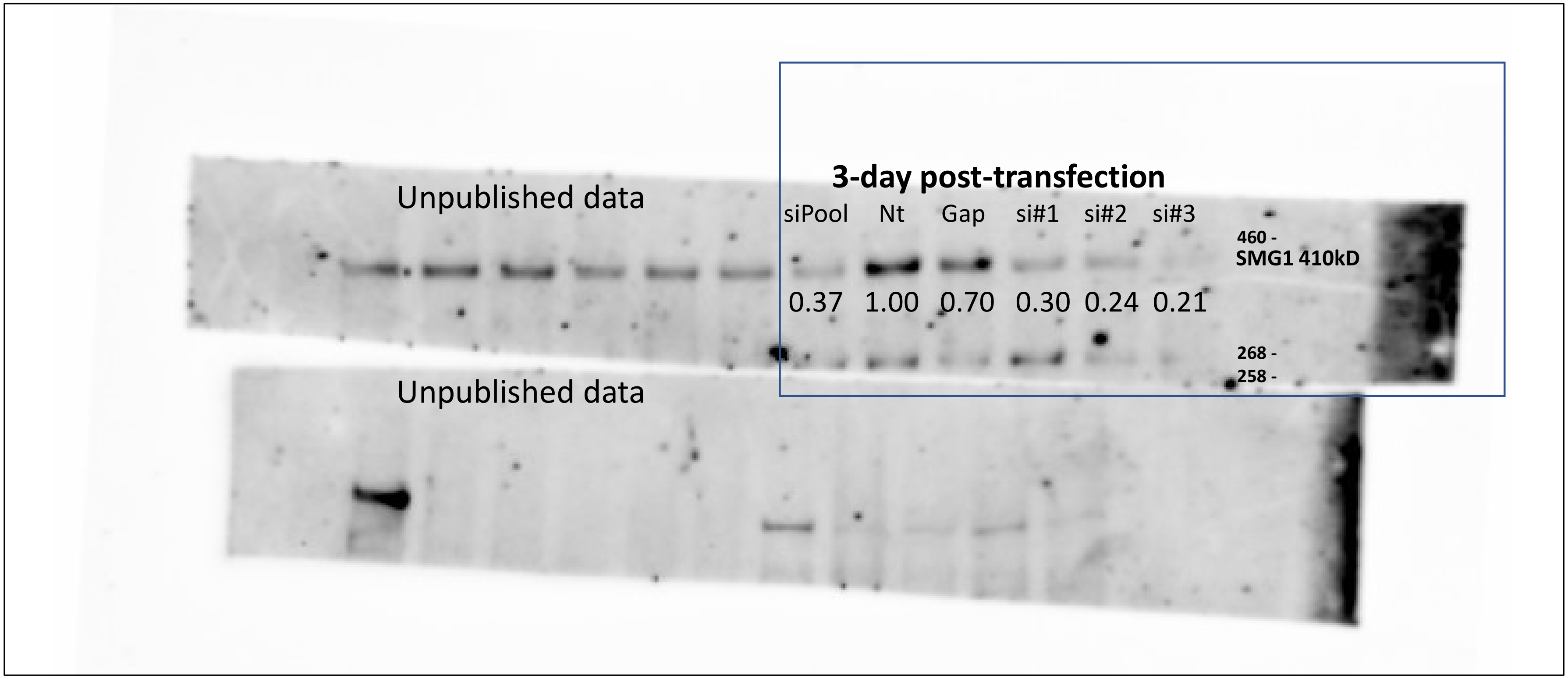
Unpublished data
3-day post-transfection
siPool
Nt
Gap
si#1
si#2
si#3
460 -
SMG1 410kD
0.37
1.00
0.70
0.30
0.24
0.21
268 -
258 -
Unpublished data

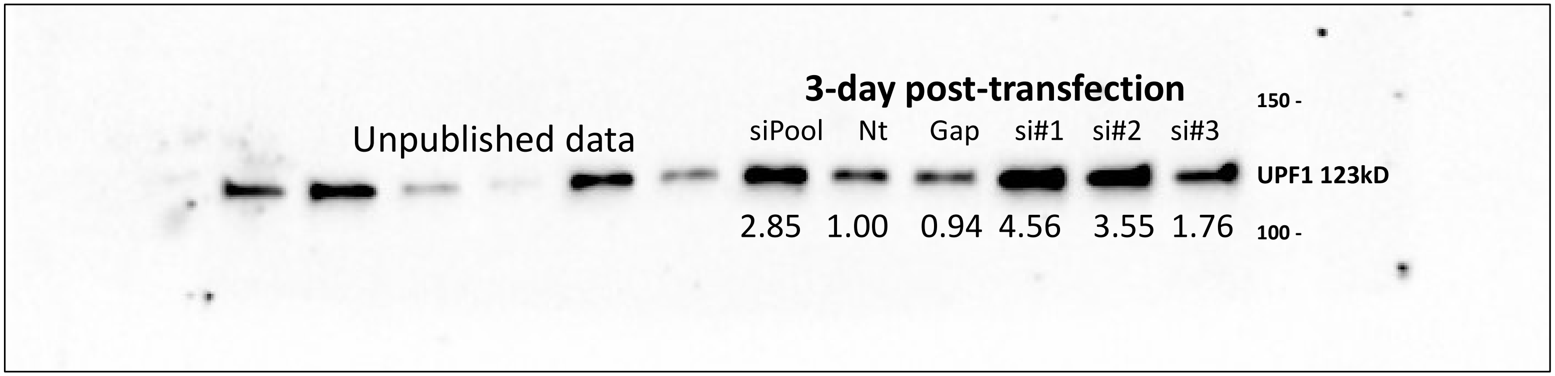
3-day post-transfection
Unpublished data
siPool
Nt
Gap
si#1
si#2
si#3
150 -
UPF1 123kD
2.85
1.00
0.94
4.56
3.55
1.76
100 -

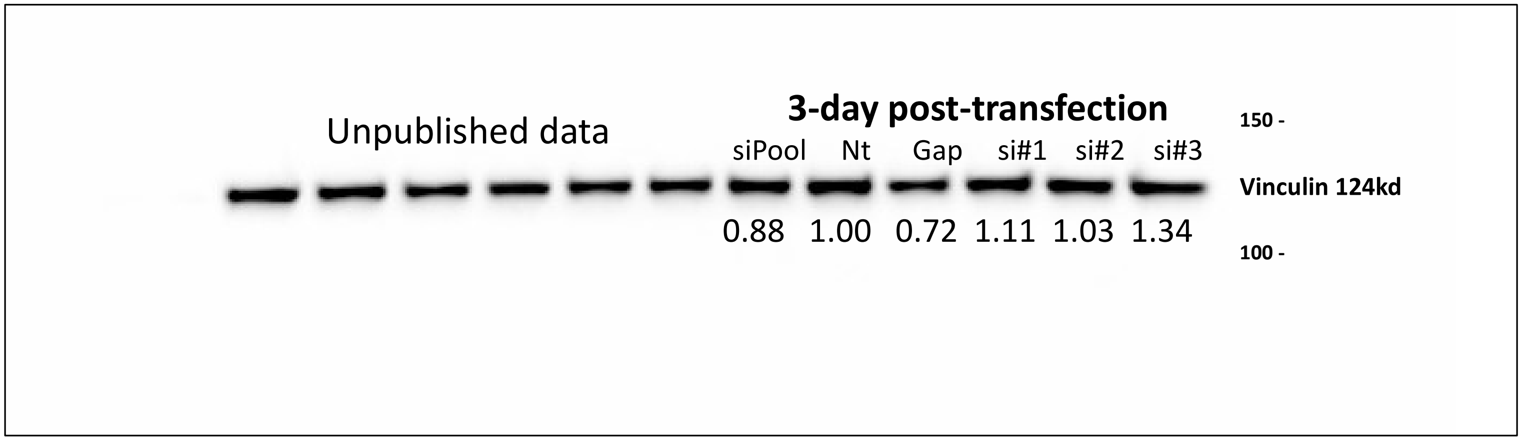
3-day post-transfection
Unpublished data
siPool
Nt
Gap
si#1
si#2
si#3
150 -
Vinculin 124kd
0.88
1.00
0.72
1.11
1.03
1.34
100 -

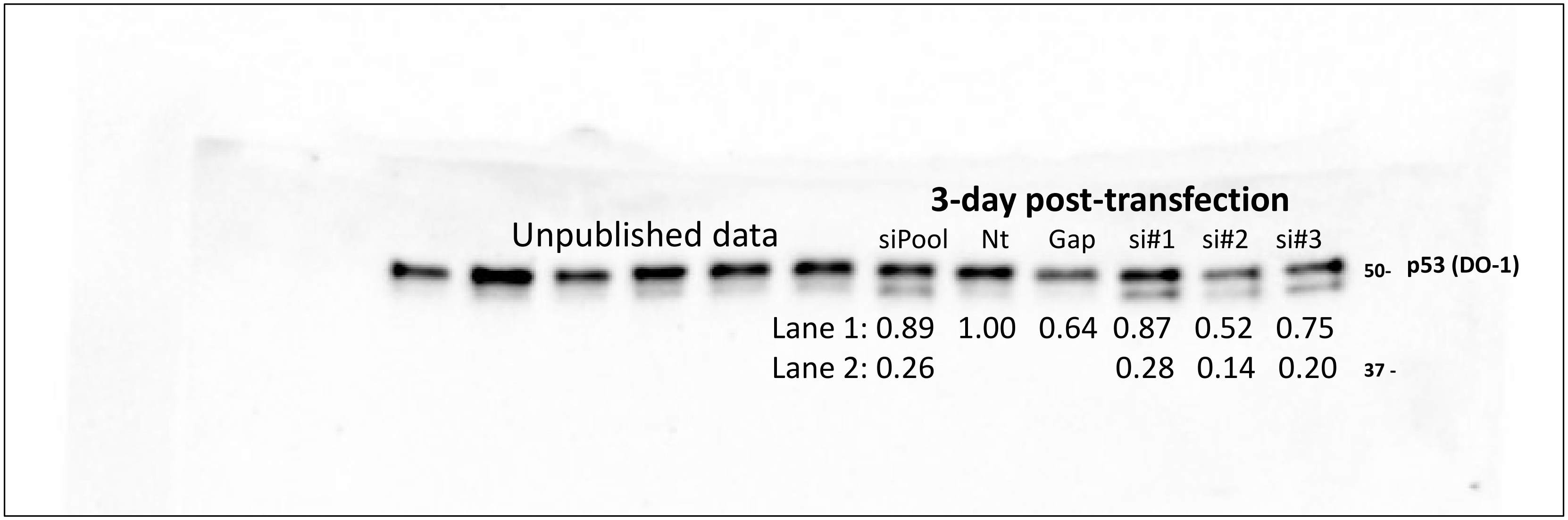
3-day post-transfection
Unpublished data
siPool
Nt
Gap
si#1
si#2
si#3
50-
p53 (DO-1)
Lane 1: 0.89 1.00 0.64 0.87 0.52 0.75
Lane 2: 0.26 0.28 0.14 0.20
37 -

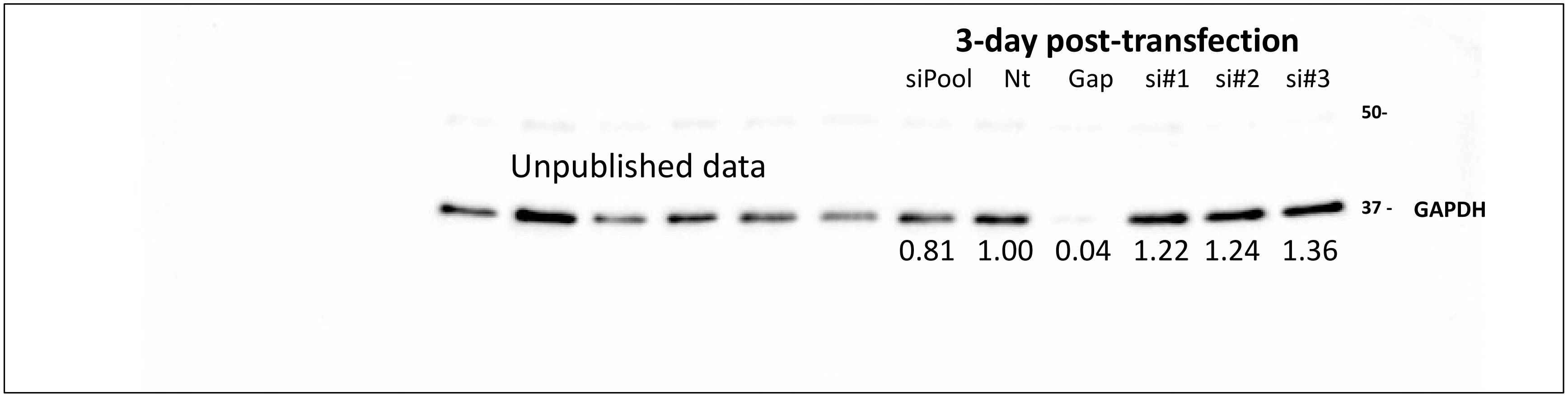
3-day post-transfection
siPool
Nt
Gap
si#1
si#2
si#3
50-
Unpublished data
37 -
GAPDH
0.81 1.00 0.04 1.22 1.24 1.36

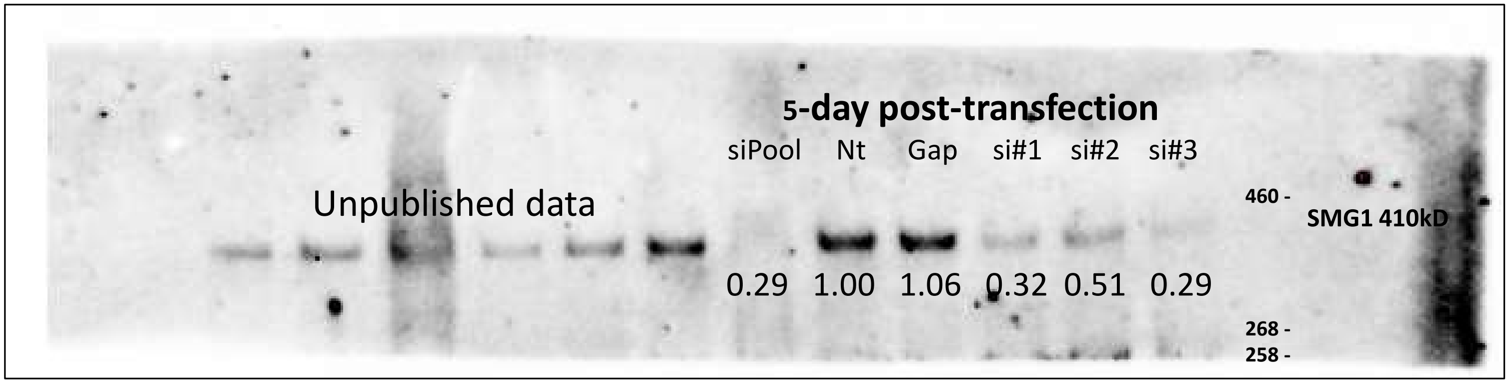
5-day post-transfection
siPool Nt Gap si#1 si#2 si#3
Unpublished data
460 -
SMG1 410kD
0.29 1.00 1.06 0.32 0.51 0.29
268 -
258 -

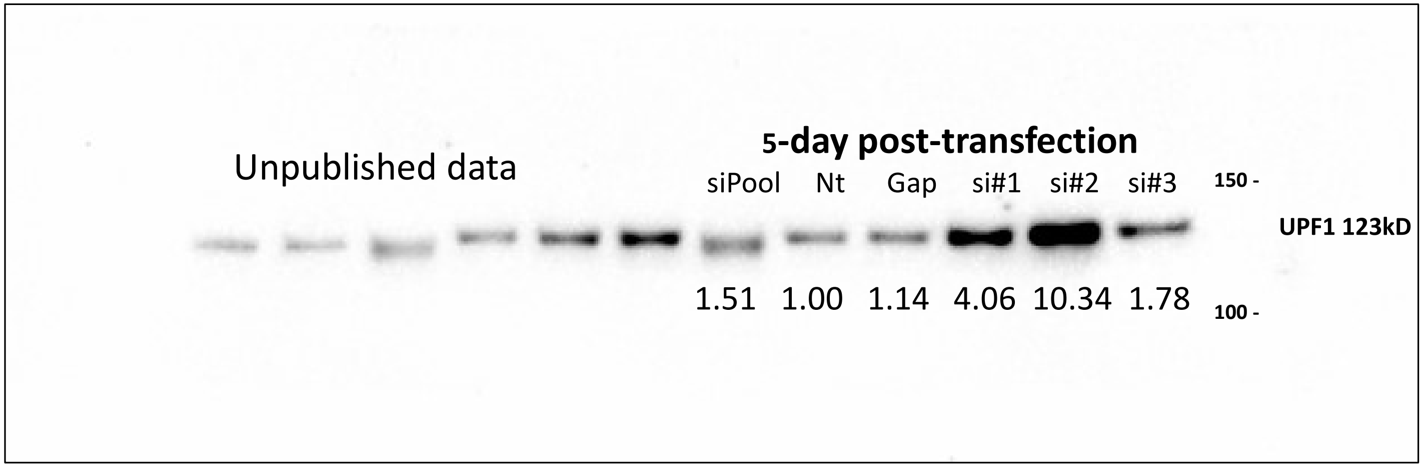
5-day post-transfection
Unpublished data
siPool Nt Gap si#1 si#2 si#3
150 -
UPF1 123kD
1.51 1.00 1.14 4.06 10.34 1.78
100 -

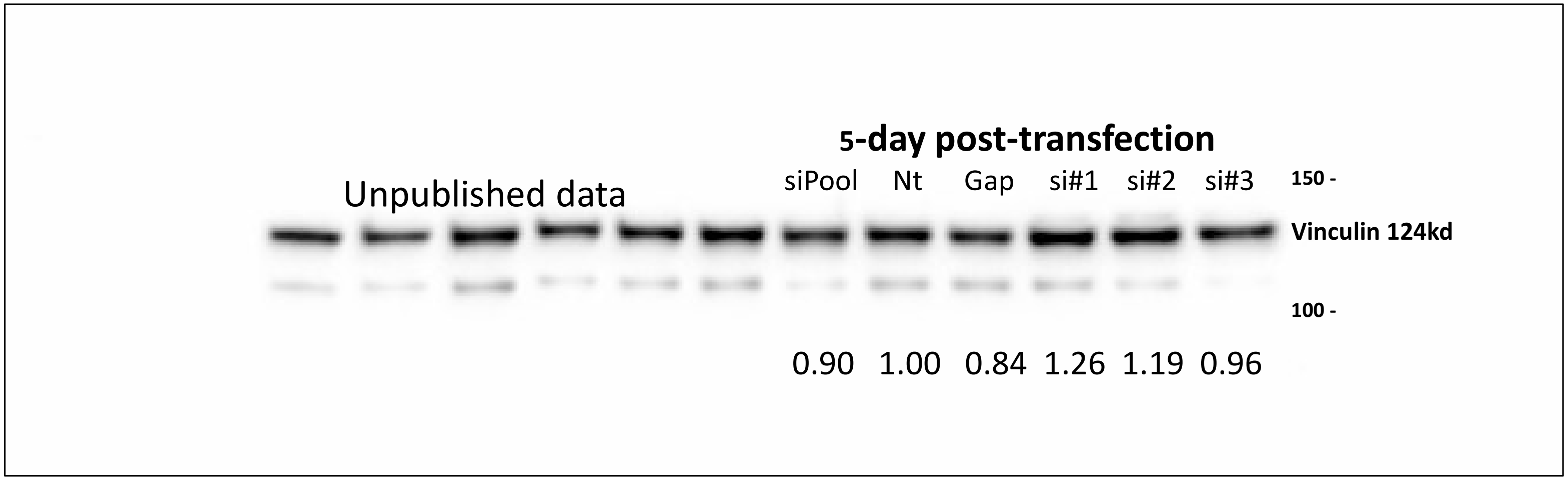

5-day post-transfection
Unpublished data
siPool Nt Gap si#1 si#2 si#3
150 -
Vinculin 124kd
100 -
0.90 1.00 0.84 1.26 1.19 0.96

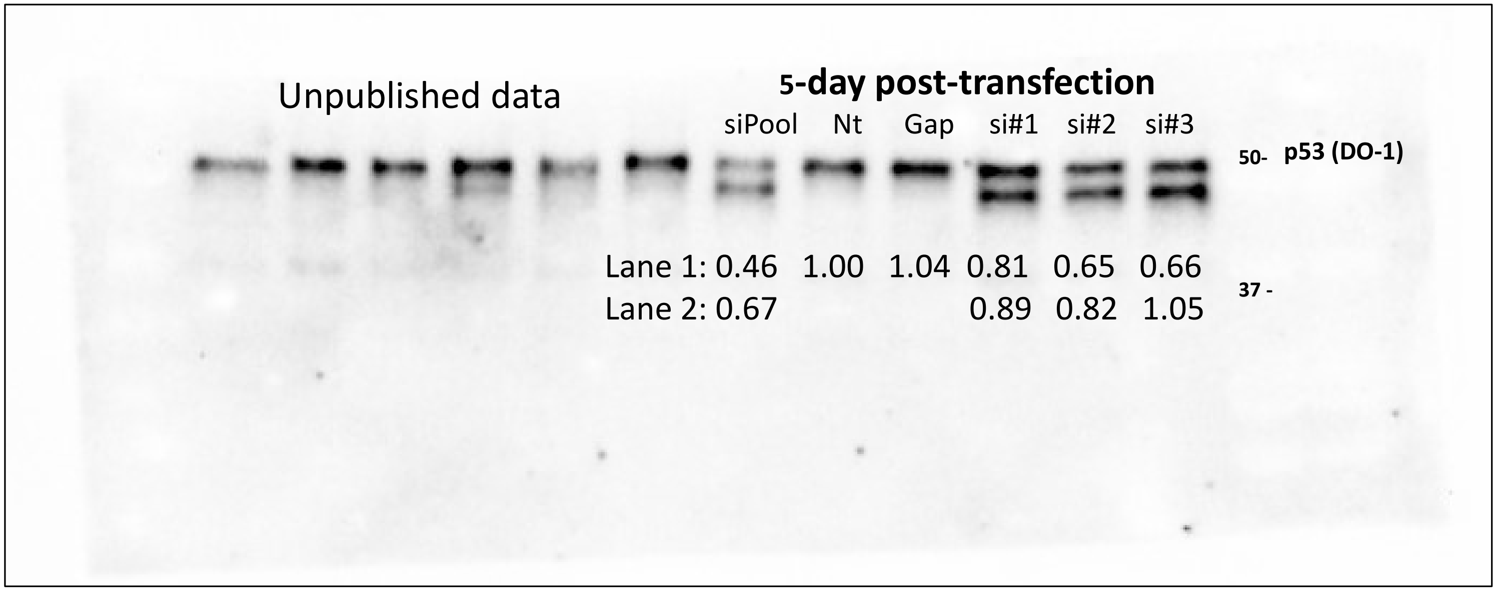

5-day post-transfection
Unpublished data
siPool Nt Gap si#1 si#2 si#3
50- p53 (DO-1)
Lane 1: 0.46 1.00 1.04 0.81 0.65 0.66
37 -
Lane 2: 0.67 0.89 0.82 1.05

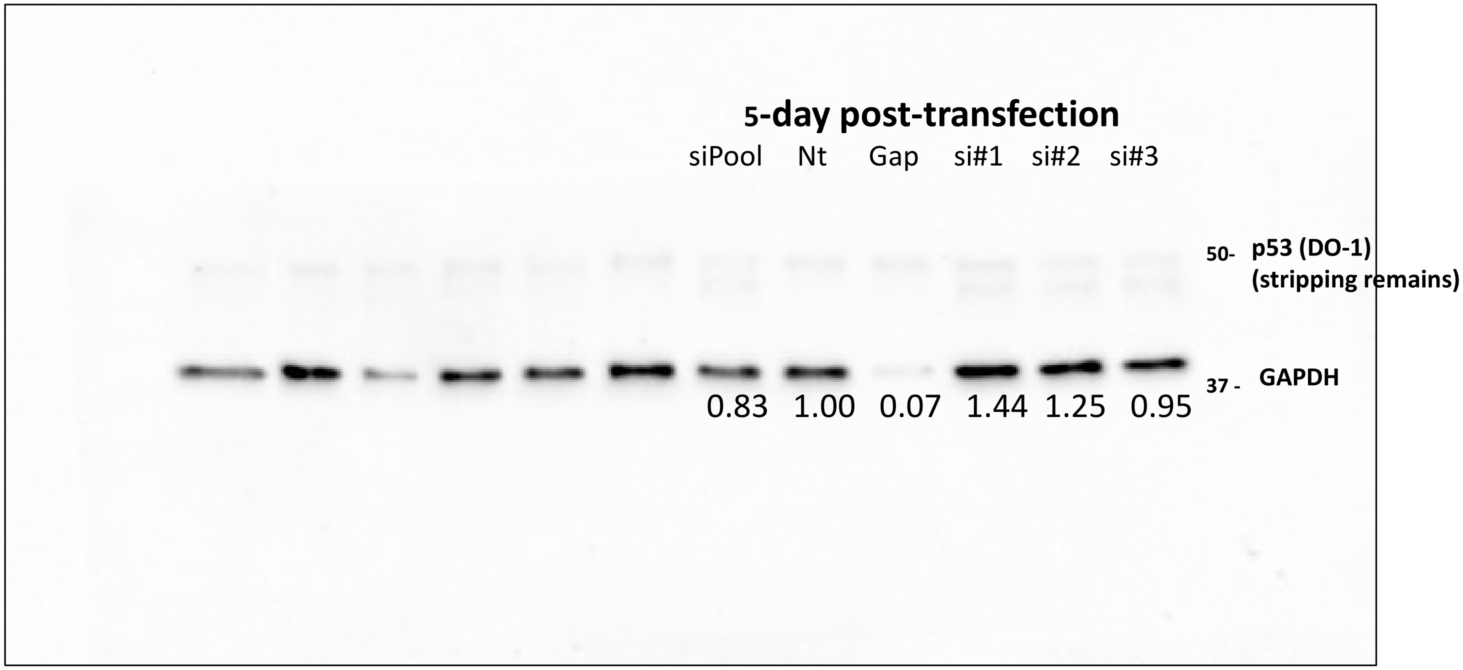
5-day post-transfection
siPool Nt Gap si#1 si#2 si#3
50-
p53 (DO-1)
(stripping remains)
37 -
GAPDH
0.83 1.00 0.07 1.44 1.25 0.95

Figure. 2D

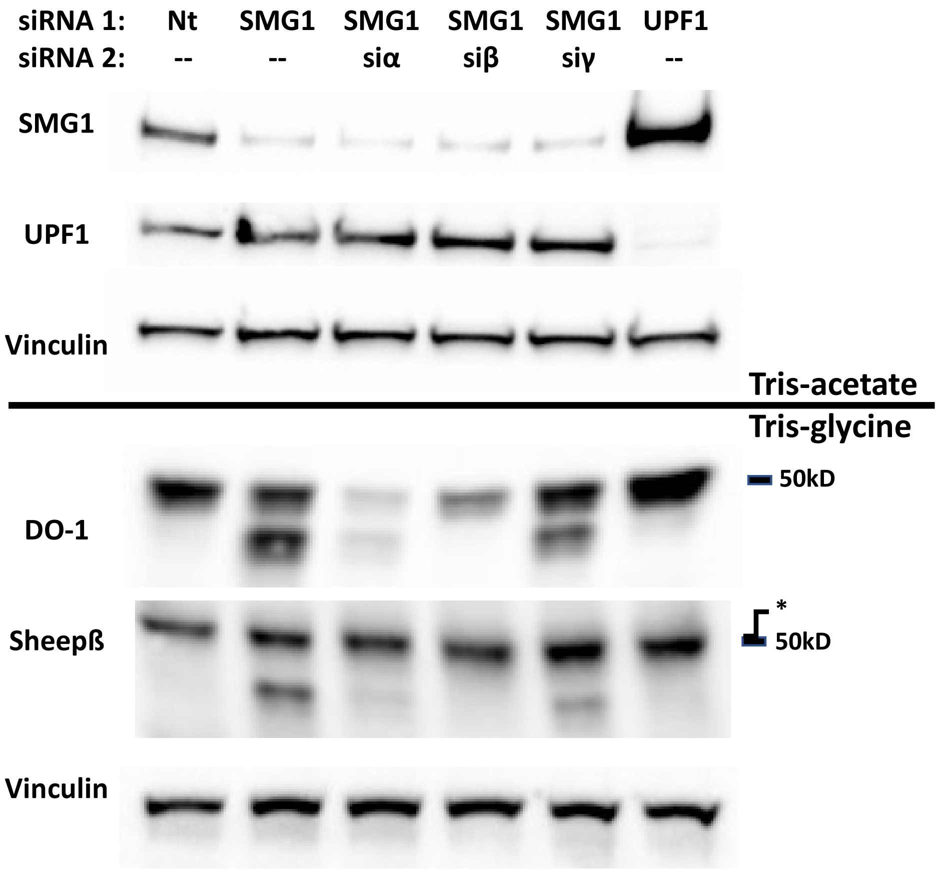

siRNA 1: Nt SMG1 SMG1 SMG1 SMG1 UPF1
siRNA 2: -- -- siα siβ siγ --
SMG1
UPF1
Vinculin
Tris-acetate
Tris-glycine
DO-1
50kD
Sheepß
*
50kD
Vinculin

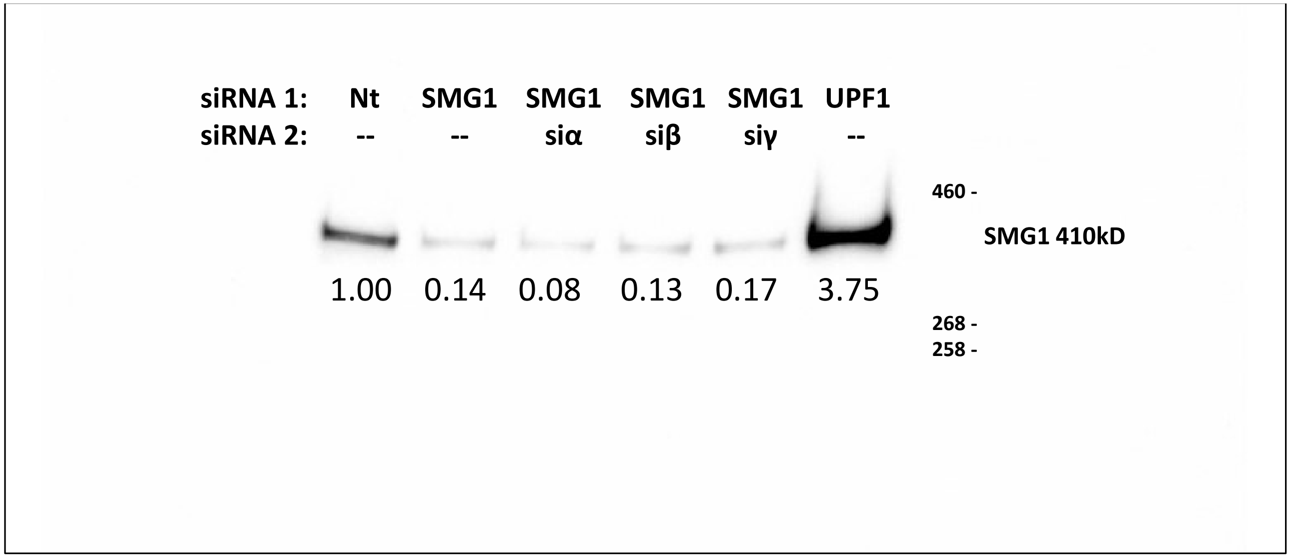
siRNA 1: Nt SMG1 SMG1 SMG1 SMG1 UPF1
siRNA 2: -- -- siα siβ siγ --
460 -
SMG1 410kD
1.00 0.14 0.08 0.13 0.17 3.75
268 -
258 -

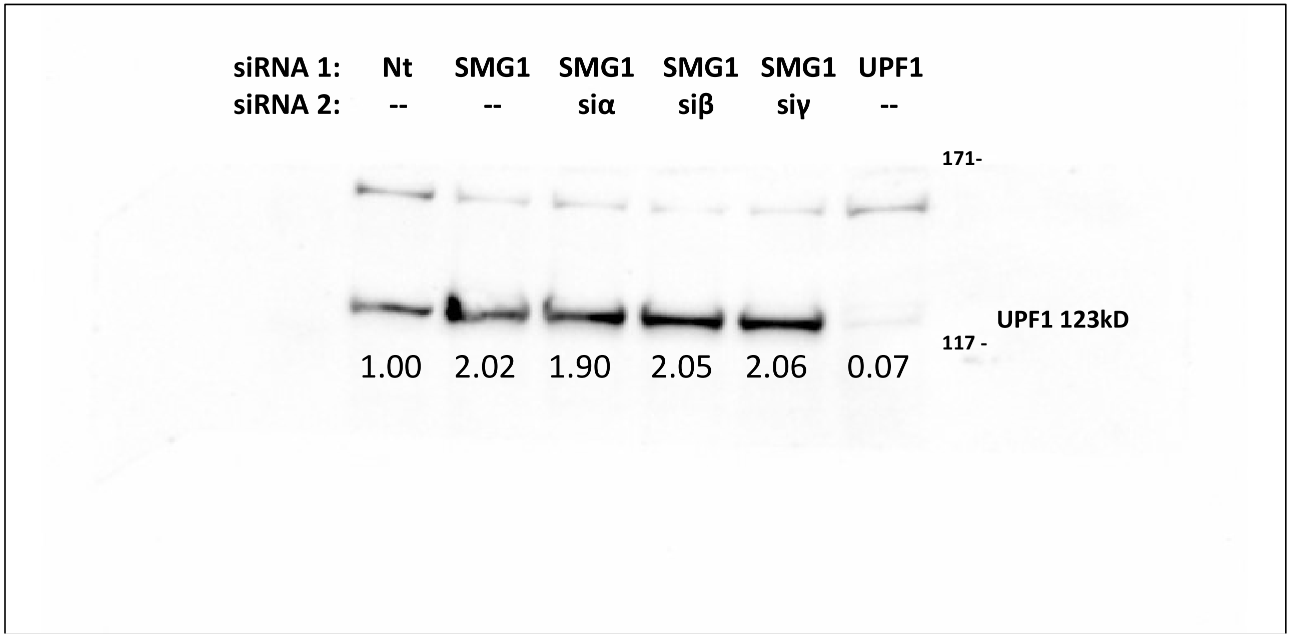
siRNA 1: Nt SMG1 SMG1 SMG1 SMG1 UPF1
siRNA 2: -- -- siα siβ siγ --
171-
UPF1 123kD
117 -
1.00 2.02 1.90 2.05 2.06 0.07

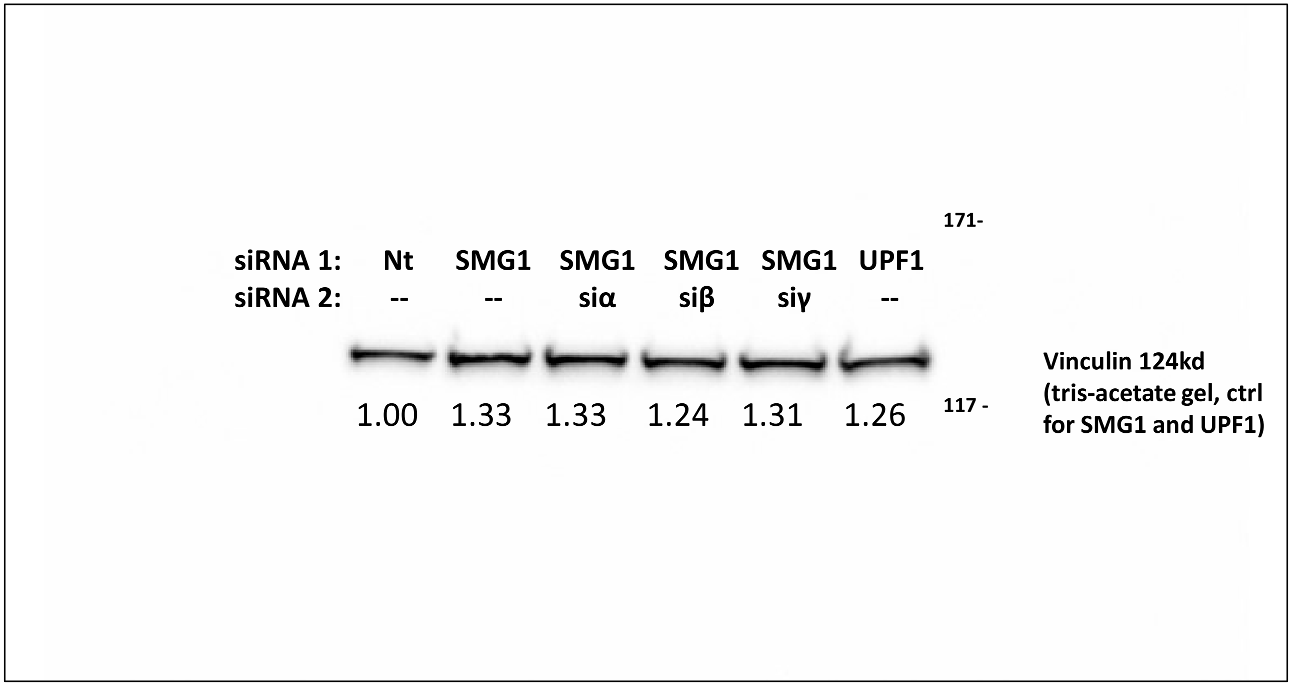
171-
siRNA 1: Nt SMG1 SMG1 SMG1 SMG1 UPF1
siRNA 2: -- -- siα siβ siγ --
Vinculin 124kd
(tris-acetate gel, ctrl
for SMG1 and UPF1)
1.00 1.33 1.33 1.24 1.31 1.26
117 -

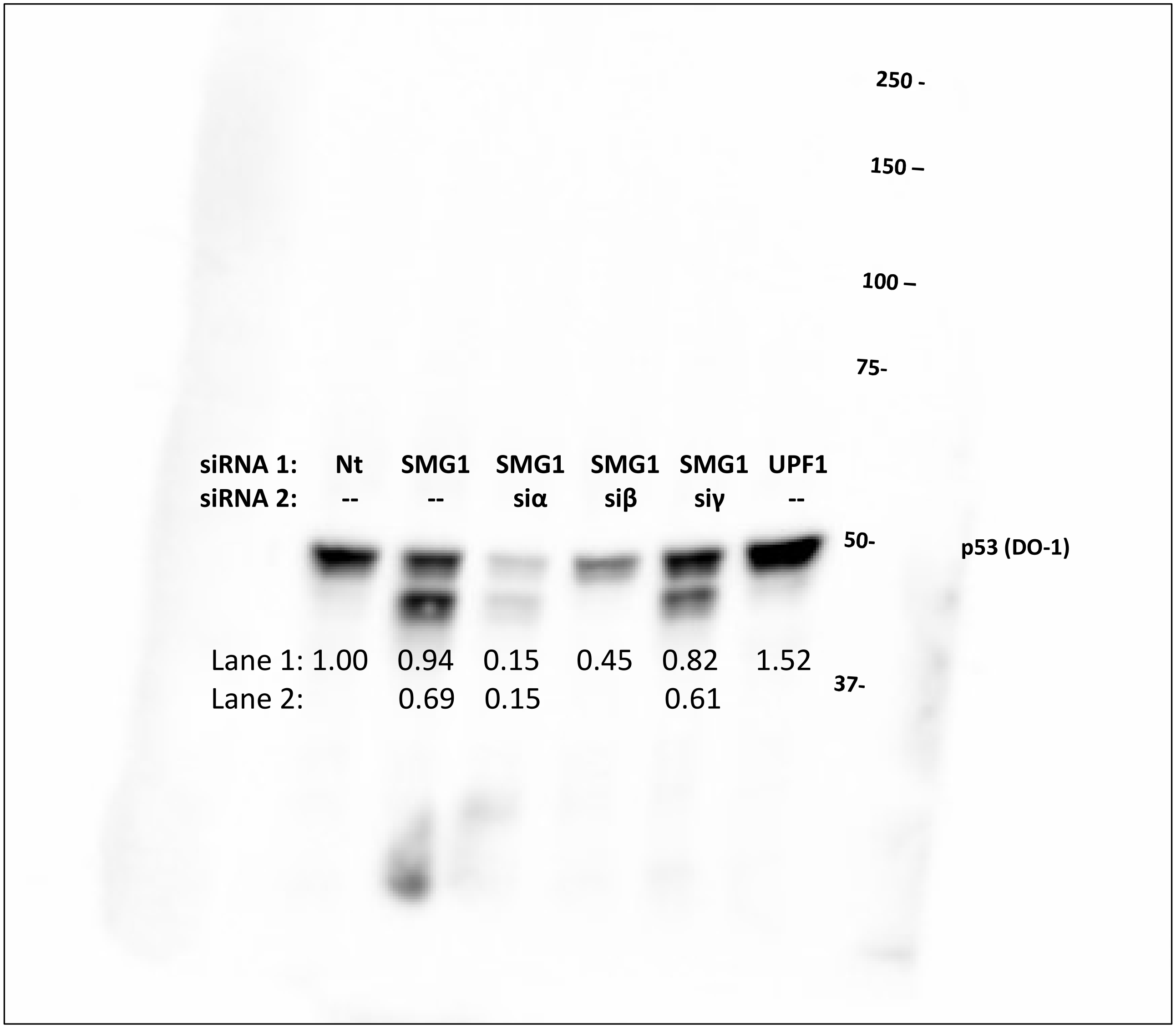
250 -
150 –
100 –
75-
siRNA 1: Nt SMG1 SMG1 SMG1 SMG1 UPF1
siRNA 2: -- -- siα siβ siγ --
50-
p53 (DO-1)
Lane 1: 1.00 0.94 0.15 0.45 0.82 1.52
Lane 2: 0.69 0.15 0.61
37-

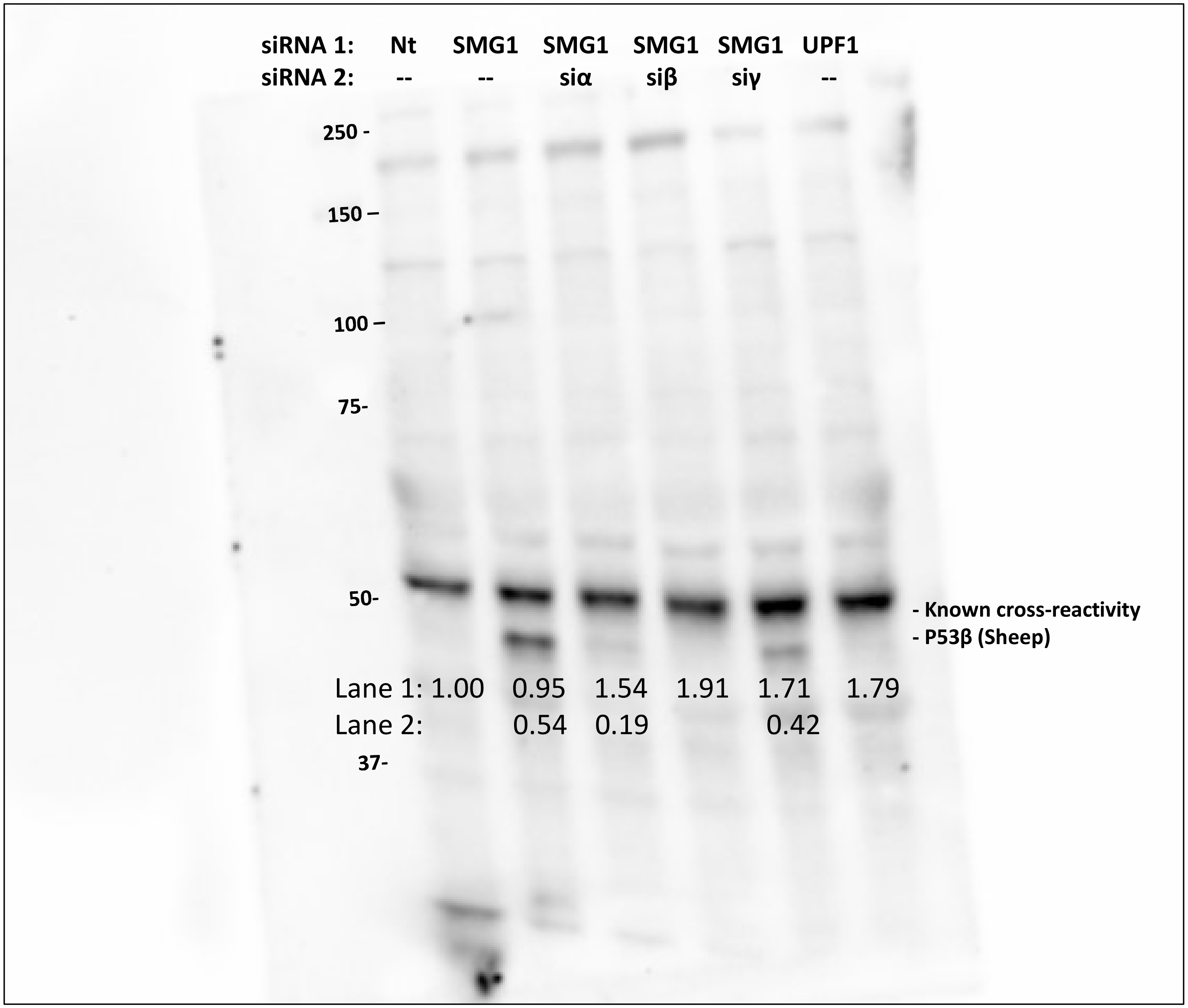
siRNA 1: Nt SMG1 SMG1 SMG1 SMG1 UPF1
siRNA 2: -- -- siα siβ siγ --
250 -
150 –
100 –
75-
50-
37-
- Known cross-reactivity
- P53β (Sheep)
Lane 1: 1.00 0.95 1.54 1.91 1.71 1.79
Lane 2: 0.54 0.19 0.42

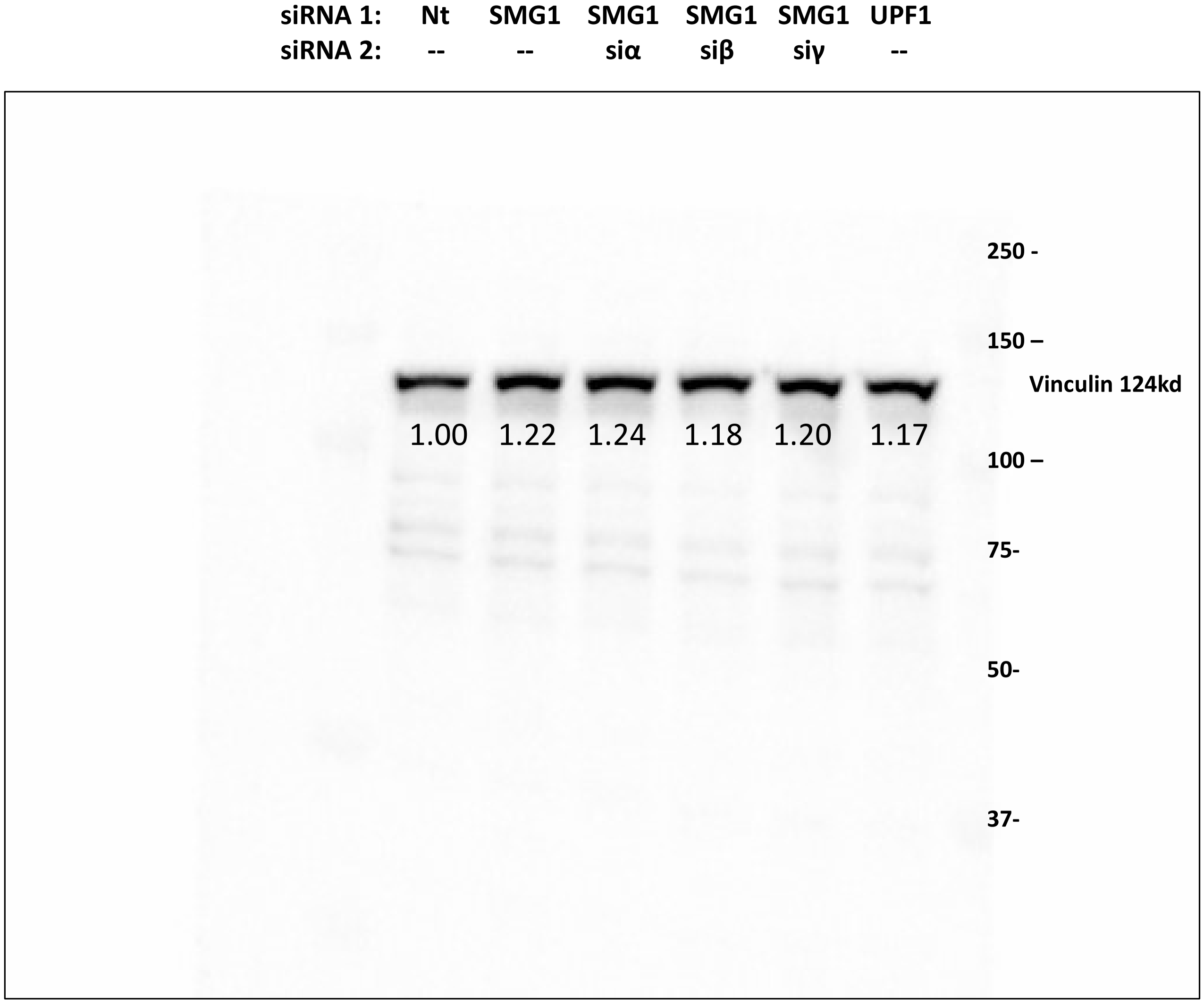
siRNA 1: Nt SMG1 SMG1 SMG1 SMG1 UPF1
siRNA 2: -- -- siα siβ siγ --
250 -
150 –
Vinculin 124kd
1.00 1.22 1.24 1.18 1.20 1.17
100 –
75-
50-
37-

Figure 3B.

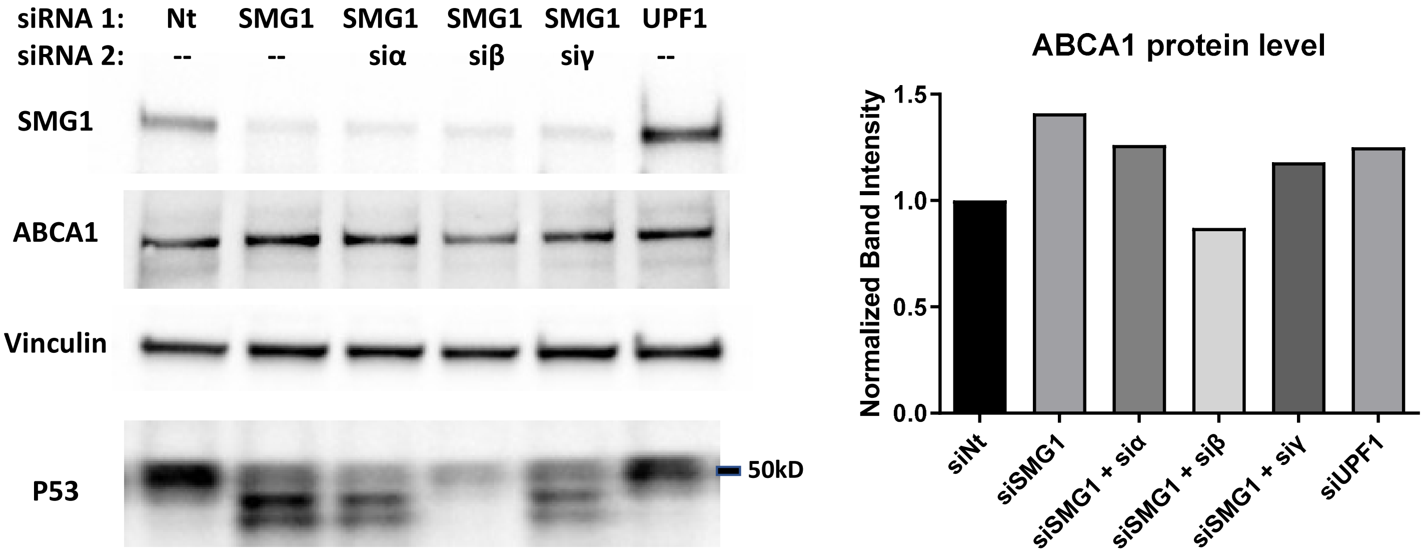
siRNA 1: Nt SMG1 SMG1 SMG1 SMG1 UPF1
siRNA 2: -- -- siα siβ siγ --
SMG1
ABCA1
Vinculin
P53
50kD
ABCA1 protein level
Normalized Band Intensity
1.5
1.0
0.5
0.0
siNt
siSMG1
siSMG1 + siα
siSMG1 + siβ
siSMG1 + siγ
siUPF1

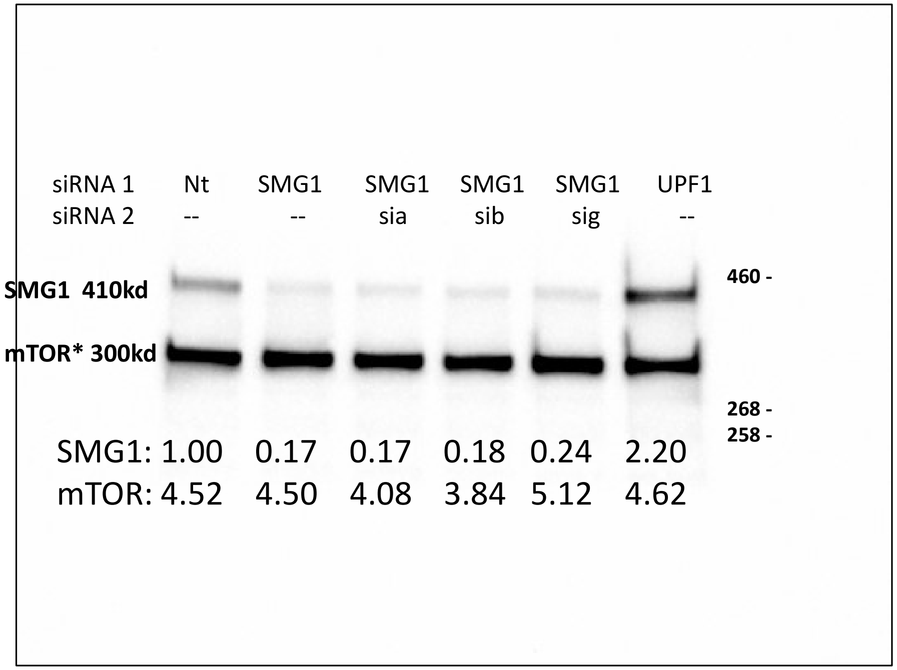

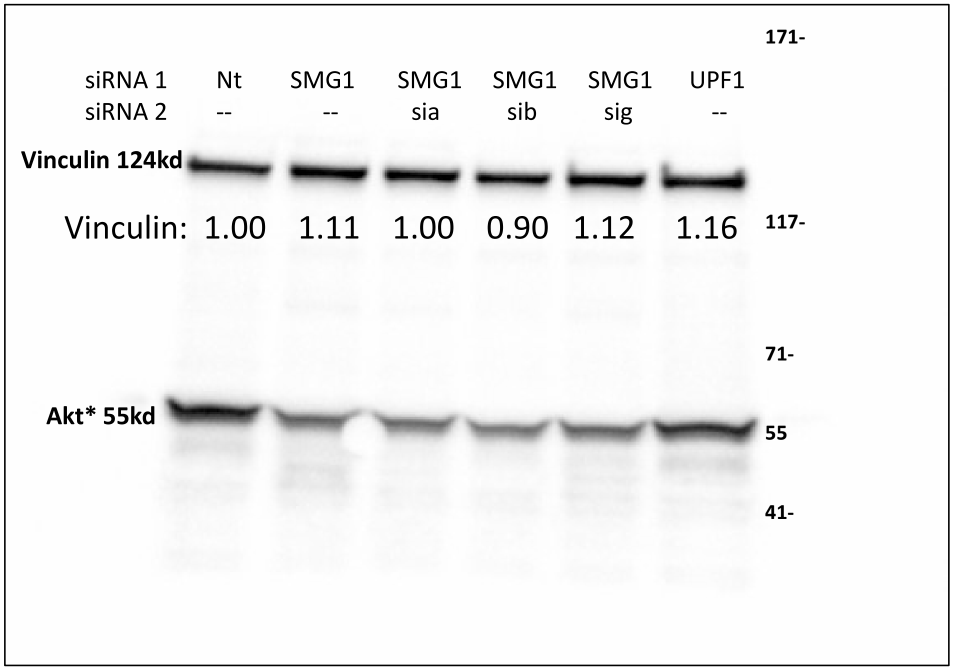

- mTOR and Akt were probed for explorational purpose but not described in the paper.
- High-quality Mouse Monoclonal antibodies that we have lots of experiences with are occasionally duplexed to save time and materials.

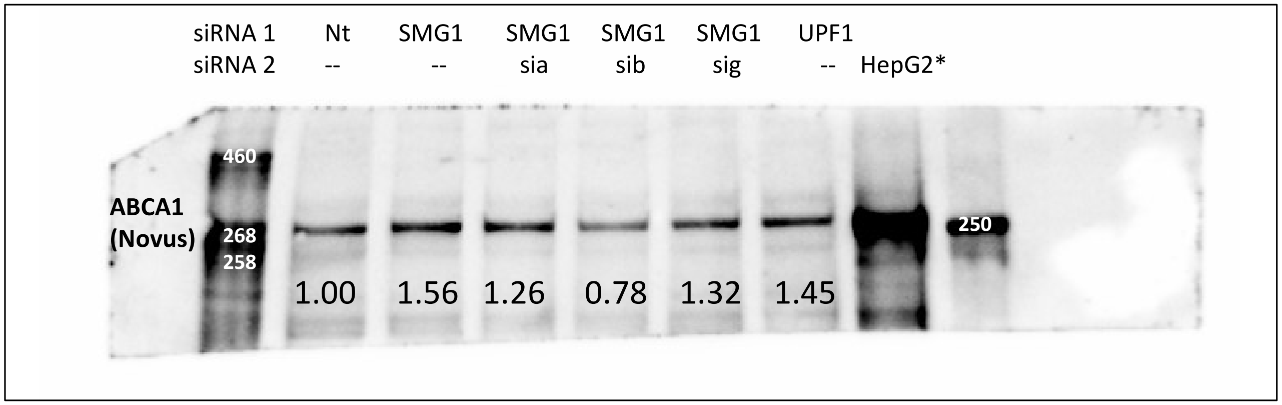

* The HepG2 lane is cropped in the article. HepG2 is a liver cancer cell line that expresses a high level of cholesterol-related genes, making it an excellent ABCA1 antibody control.

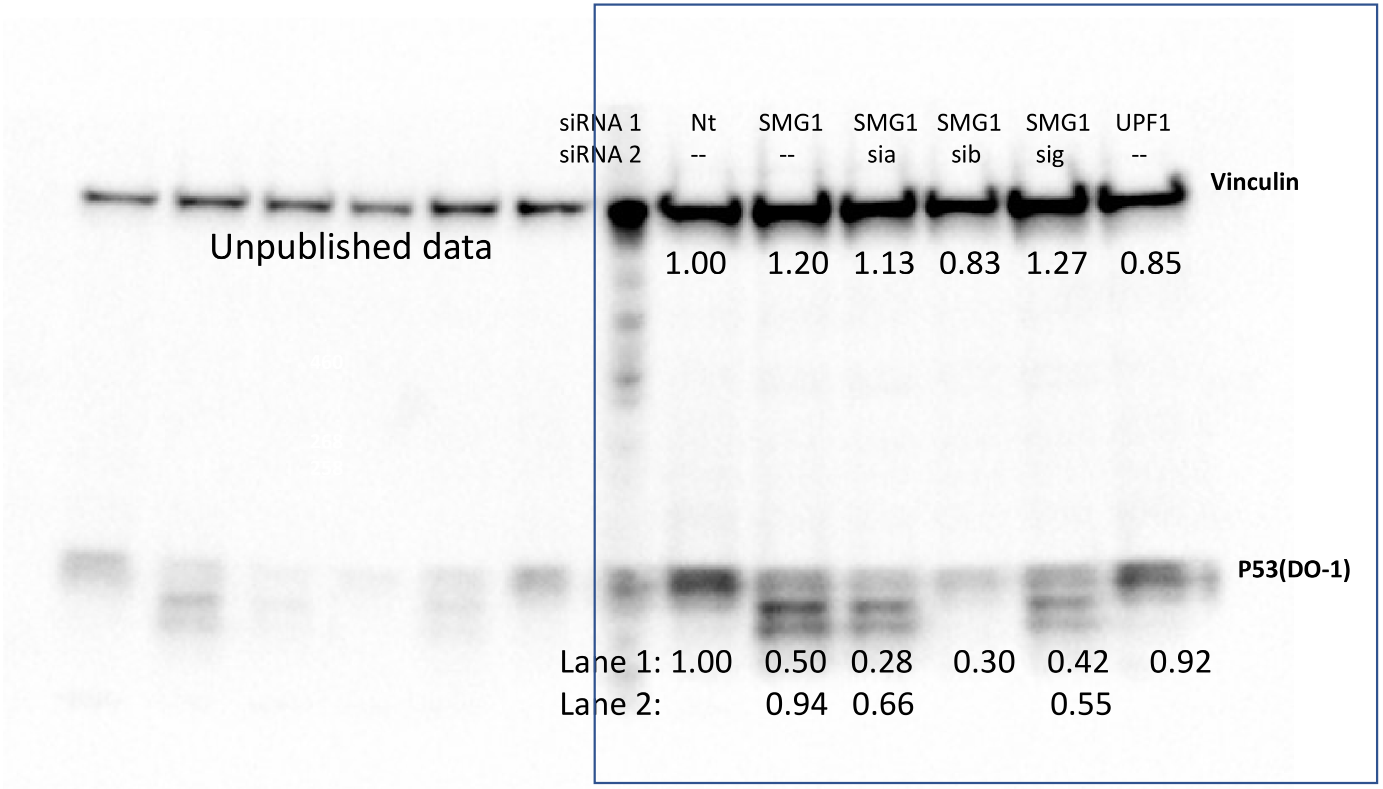
siRNA 1
siRNA 2
Nt
--
SMG1
--
SMG1
sia
SMG1
sib
SMG1
sig
UPF1
--
Vinculin
Unpublished data
1.00 1.20 1.13 0.83 1.27 0.85
P53(DO-1)
Lane 1: 1.00 0.50 0.28 0.30 0.42 0.92
Lane 2: 0.94 0.66 0.55

Figure. 4C

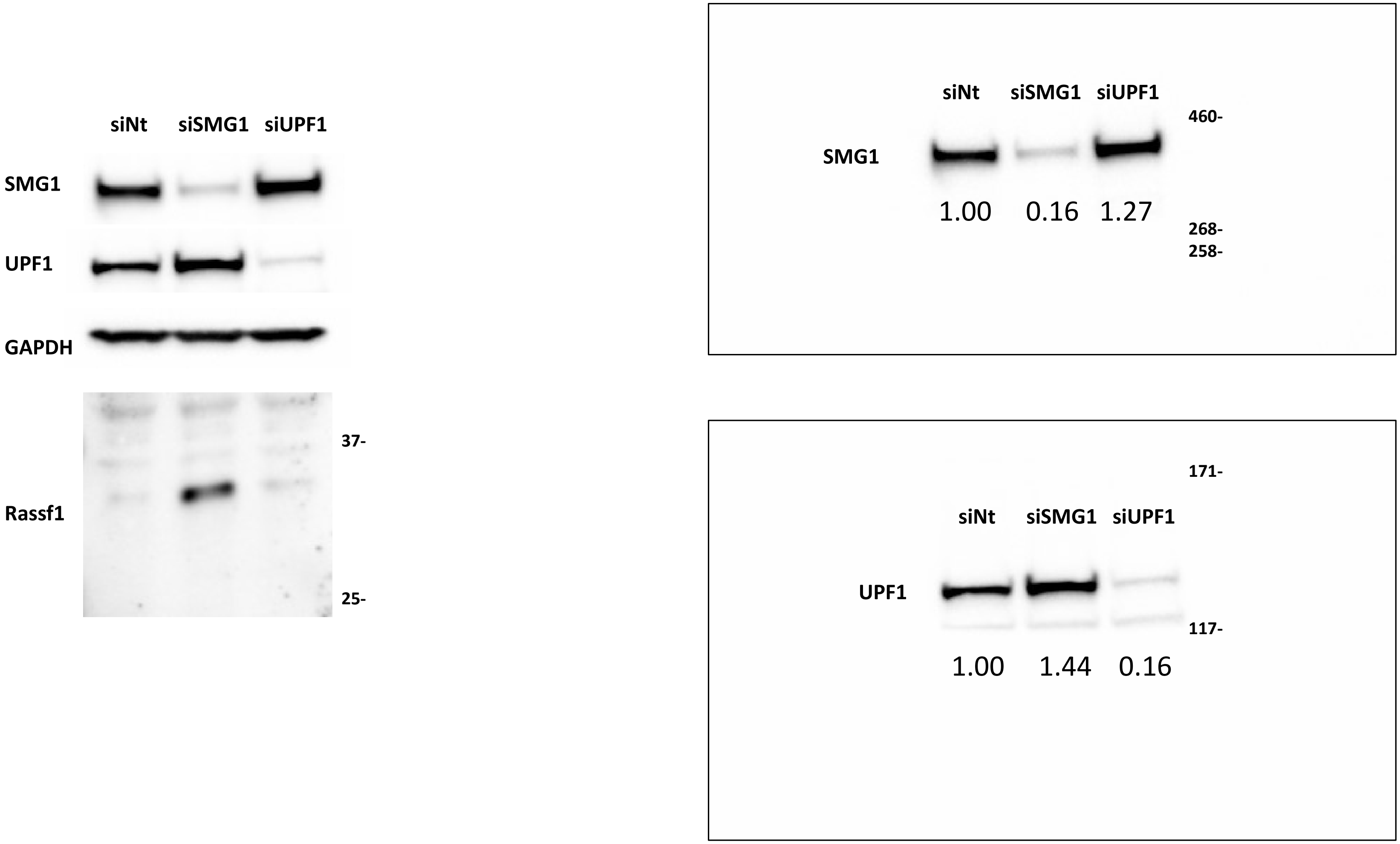
siNt
siSMG1
siUPF1
SMG1
UPF1
GAPDH
Rassf1
37-
25-
siNt
siSMG1
siUPF1
460-
SMG1
1.00
0.16
1.27
268-
258-
171-
siNt
siSMG1
siUPF1
UPF1
117-
1.00
1.44
0.16

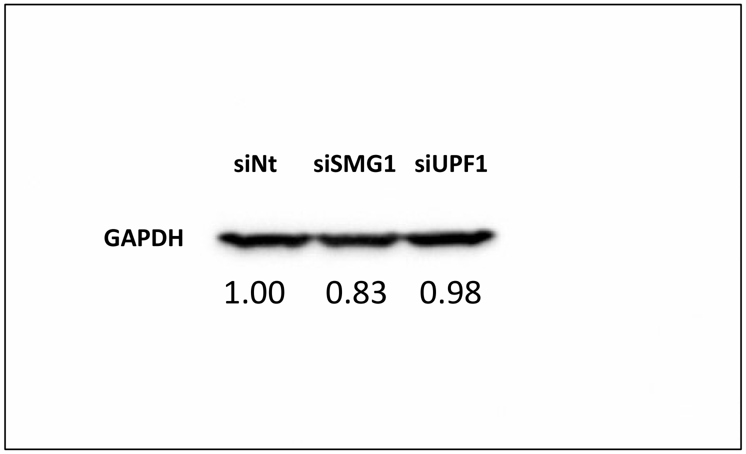

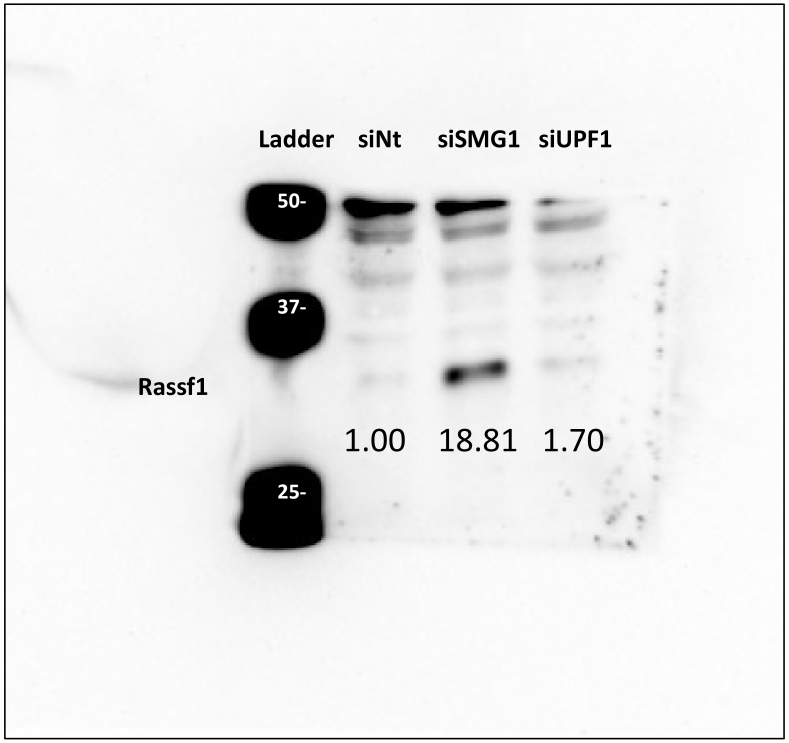

* To more accurately locate rassf1 band considering it may have isoforms, we used Precision Plus Protein unstained standards and StrepTactin-HRP conjugate. This blot is 5-min exposure time, hence the ladder lane is overexposed.

Figure. S4

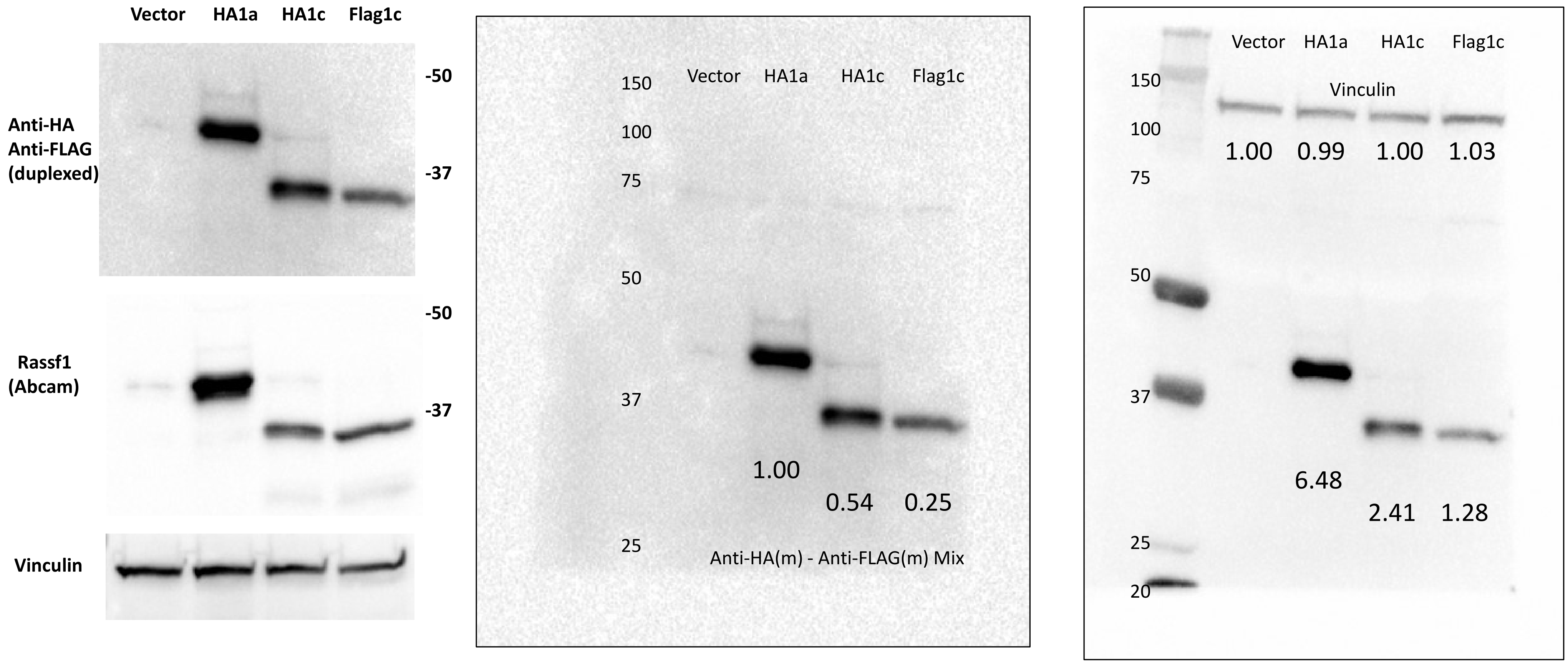

Vector
HA1a
HA1c
Flag1c
-50
Anti-HA
Anti-FLAG
(duplexed)
-37
-50
Rassf1
(Abcam)
-37
Vinculin
150
Vector
HA1a
HA1c
Flag1c
100
75
50
37
1.00
0.54
0.25
25
Anti-HA(m) - Anti-FLAG(m) Mix
Vector
HA1a
HA1c
Flag1c
150
Vinculin
100
1.00
0.99
1.00
1.03
75
50
37
6.48
2.41
1.28
25
20

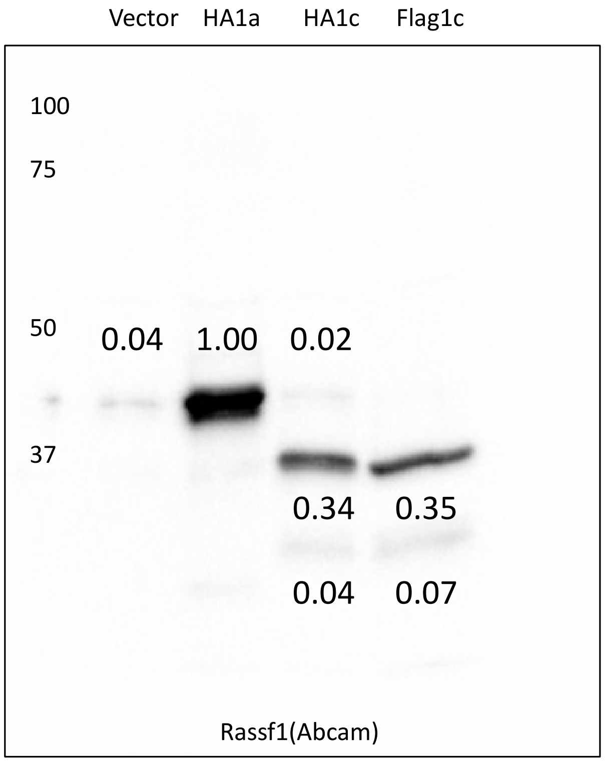
Vector
HA1a
HA1c
Flag1c
100
75
50
37
0.04
1.00
0.02
0.34
0.35
0.04
0.07
Rassf1(Abcam)

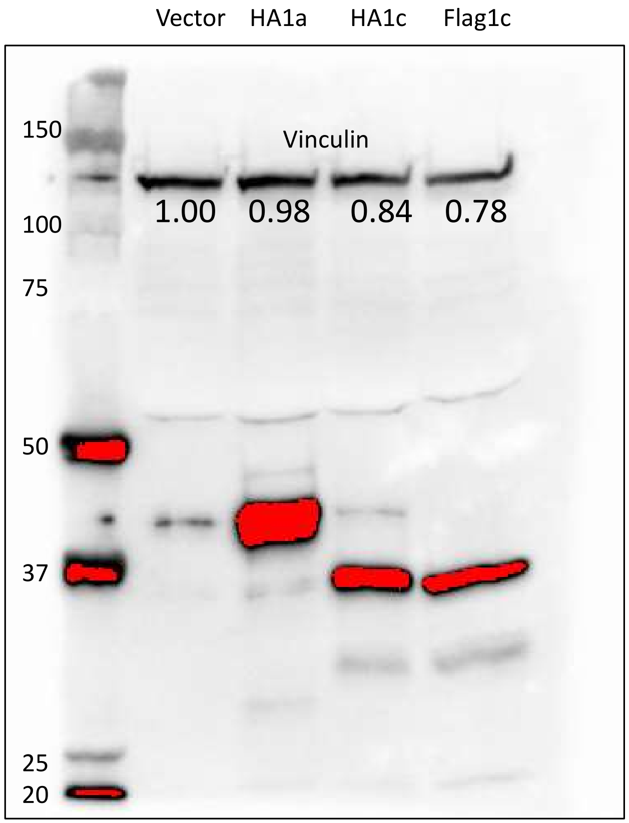
Vector
HA1a
HA1c
Flag1c
150
Vinculin
1.00
0.98
0.84
0.78
100
75
50
37
25
20
